# Altered β-Adrenergic System, Cardiac Dysfunction, and Lethal Arrhythmia in a Rat Model of Metabolic Syndrome

**DOI:** 10.3390/ijms26167989

**Published:** 2025-08-19

**Authors:** Rommel Sánchez-Hernández, Daphne E. Cruz-Villarreal, Alejandro Silva-Palacios, Alejandra-María Zúñiga-Muñoz, Elizabeth Soria-Castro, Carlos Sánchez-Garibay, Cecilia Zazueta, J. Alberto Olivares-Reyes, José-Antonio Arias-Montaño, Angélica Rueda

**Affiliations:** 1 Department of Physiology, Biophysics and Neurosciences, Center for Research and Advanced Studies (Cinvestav) of the National Polytechnic Institute, Av. IPN 2508, San Pedro Zacatenco, Mexico City 07300, Mexico; rommel.sanchez@cinvestav.mx; 2 Department of Biochemistry, Center for Research and Advanced Studies (Cinvestav) of the National Polytechnic Institute, Av. IPN 2508, San Pedro Zacatenco, Mexico City 07300, Mexico; daphne.cruz@cinvestav.mx (D.E.C.-V.); jolivare@cinvestav.mx (J.A.O.-R.); 3 Department of Cardiovascular Biomedicine, National Institute of Cardiology Ignacio Chávez, Juan Badiano 1, Belisario Domínguez Secc 16, Tlalpan, Mexico City 14080, Mexico; alejandro.silva@cardiologia.org.mx (A.S.-P.); alejandra.zuniga@cardiologia.org.mx (A.-M.Z.-M.); elizabeth.soria@cardiologia.org.mx (E.S.-C.); ana.zazueta@cardiologia.org.mx (C.Z.); 4 Department of Neuropathology, National Institute of Neurology and Neurosurgery Manuel Velasco Suárez, Insurgentes Sur 3877, La Fama, Tlalpan, Mexico City 14269, Mexico; carlos.s.garibay@live.com.mx

**Keywords:** metabolic syndrome, heart, isoproterenol, β-adrenoceptors, β-adrenergic signaling, arrhythmia, cardiometabolic disease

## Abstract

Metabolic syndrome (MetS) is a worldwide problem affecting at least one-third of the population. MetS patients have increased cardiovascular risk associated with an abnormal β-adrenergic response; however, it is not clear how MetS affects the cardiac β-adrenergic system. We analyzed cardiac function and the β-adrenergic response in an experimental model of MetS in rats by recording pressure–volume (PV) loops via an open-chest approach and performed a biochemical characterization of the cardiac β-adrenergic system through ELISA, radioligand binding assays, and Western blotting. Microscopy was employed to evaluate cardiac hypertrophy, fibrosis, and ultrastructure. MetS rats exhibited cardiac dysfunction, evidenced by a reduced cardiac output and ejection fraction, not explained by heart hypertrophy or fibrosis. MetS rats also had an elevated susceptibility to lethal arrhythmia following intra-cardiac administration of the non-selective β-adrenergic agonist isoproterenol, suggesting alterations in the β-adrenergic system. The total serum adrenaline and noradrenaline levels were higher in the MetS animals than those in the control group. The radioligand binding assays indicated no change in the βAR density; however, the Western blot analyses revealed decreased levels of Gα_s_ proteins and β-arrestin 1, but increased β_2_AR and Gα_i_ protein levels. This study contributes to our understanding of how MetS can alter cardiac function, raising the risk of lethal arrhythmia induced by the β-adrenergic (fight or flight) response and underscores the relevance of therapeutically targeting MetS before its pathological progression toward cardiomyopathy.

## 1. Introduction

Metabolic syndrome (MetS) affects at least one-third of the global population and comprises a cluster of alterations, namely, central obesity, elevated triglyceride levels, low levels of high-density lipoprotein cholesterol (HDL-C), high blood pressure, and dysglycemia, ultimately increasing the risk of type 2 diabetes mellitus (DM2) and cardiovascular diseases (CVDs) [1,2].

MetS patients have an increased firing rate of sympathetic fibers [3], augmented basal heart rate [4], over-excretion of urine noradrenaline [5], and elevated plasma noradrenaline levels [6], suggesting overactivation of the sympathetic nervous system (SNS). During stressful situations, the SNS increases cardiac performance to meet the oxygen demand; however, a hyperadrenergic state impairs the physiological cardiac response to adrenaline and noradrenaline and may increase CVD risk by diminishing the cardiac adrenergic reserve, leading to inefficient heart performance during stress or exercise [7].

The sympathetic nerve terminals synthesize and release noradrenaline, while chromaffin cells of the adrenal glands synthesize and release adrenaline, and both transmitters exert their effects through the activation of their cognate receptors (adrenoceptors) [8]. There are three families of adrenoceptors (α_1_, α_2_, and β), and all three β-adrenergic receptor (βAR) subtypes (β_1_AR, β_2_AR, and β_3_AR) are expressed in the heart [9,10].

βARs belong to the superfamily of G protein-coupled receptors (GPCRs) and activate Gα_s_ proteins, leading to the synthesis of the second messenger 3′-5′-cyclic adenosine monophosphate (cAMP), which in turn activates two kinases, protein kinase A (PKA) and, via the exchange protein activated by cAMP (Epac), Ca^2+^-Calmodulin Kinase type II (CaMKII) [11], leading to the phosphorylation of several proteins that participate in cardiac excitation–contraction coupling (ECC), such as intracellular Ca^2+^ channel/ryanodine receptors (RyRs) and phospholamban (PLN). β_2_ARs can also couple to Gα_i/o_ proteins, decreasing cAMP levels and PKA activity [12].

As in humans, alterations in the β-adrenergic system have been reported for experimental models of obesity and MetS. For instance, rats with MetS induced by the consumption of 32% sucrose in their drinking water have diminished protein expression of β_1_ARs and β_2_ARs, and increased PKA activity in left ventricle (LV) homogenates [13]. Mice with obesity induced by a high-fat diet show β_2_AR desensitization due to the augmented phosphorylation of Ser^261/262^ and Ser^355/356^ by PKA and the GPCR kinase 2 (GRK2), respectively [14]. However, there are still discrepancies regarding the altered β-adrenergic response in these pre-diabetic conditions. In particular, obesity induced in rats by high-fat-diet consumption or sucrose feeding did not induce changes in cardiac βAR expression [13] or PKA expression and function [13,14]. Thus, despite the evidence that MetS leads to the overactivation of the SNS [7], it remains unclear how the cardiac β-adrenergic system is altered.

In the experimental model of MetS induced in Wistar rats by the intake of sucrose (30%) in drinking water for four months, animals developed obesity and showed high levels of triglycerides, decreased high-density lipoprotein cholesterol (HDL-C), glucose intolerance, and hyperinsulinemia, along with insulin resistance [15,16]. We reported previously that cardiomyocytes isolated from MetS rats exhibit augmented diastolic Ca^2+^ leakage, exacerbated by stimulation with the non-selective βAR agonist isoproterenol, indicating alterations in the cardiac β-adrenergic response [16]. The autonomous activation of CaMKII by autophosphorylation explains these results to some extent, through the phosphorylation of RyR2 at Ser^2814^, leading to abnormal Ca^2+^ leakage from the sarcoplasmic reticulum [17]; however, additional understanding of the detrimental changes induced by MetS in the main components of the cardiac β-adrenergic signaling pathway, upstream from the well-known effectors PKA and CaMKII, is needed, and this study addressed this imperative.

## 2. Results

### 2.1. Characterization of the MetS Model

**Figure 1:**
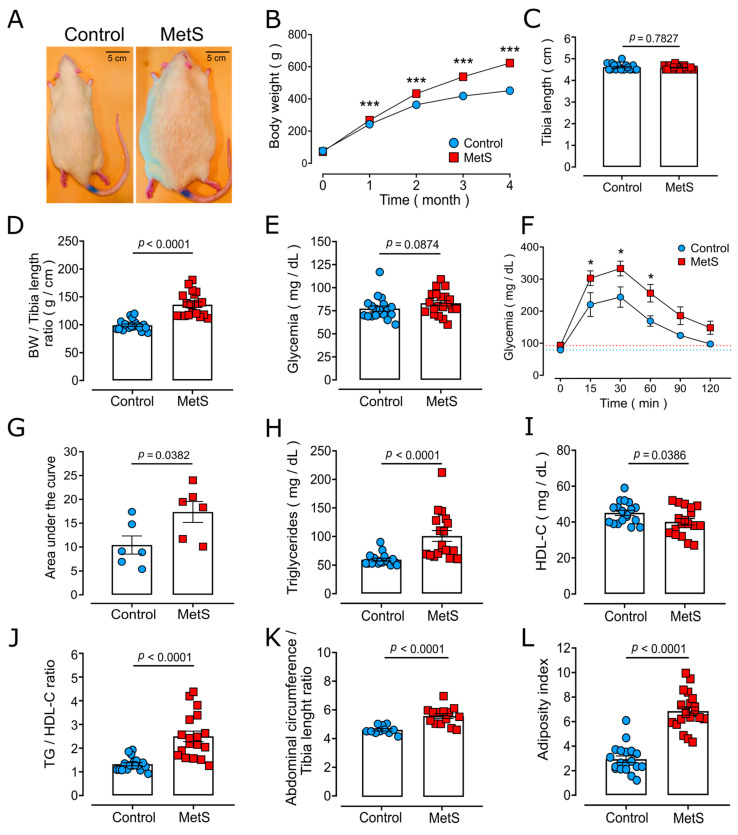
**Chronic sugar consumption leads to the development of metabolic syndrome (MetS) characteristics in male Wistar rats.** Data points represent individual determinations, except for panels B and F. (**A**) Representative pictures of control and MetS rats. (**B**) Time course of body weight. (**C**) Tibia length. (**D**) Body weight (BW)/tibia length ratio at the end of the experimental procedure. (**E**) Fasting glycemia. (**F**,**G**) Glucose tolerance test: time course (**F**) and area under the curve (**G**). Values are from 6 rats per group. The dotted lines in panel F indicate initial glycemia values (0 min) for control (blue) and MetS (red) animals. (**H**–**J**) Fasting values of (**H**) triglycerides (TGs), (**I**) high-density lipoprotein cholesterol (HDL-C), and the (**J**) TG/HDL-C ratio. (**K**,**L**) Values for abdominal circumference/tibia length ratio (**K**) and adiposity index (**L**). Data are means ± standard errors of the means (SEMs) from N = 19 control rats (blue circles) and N = 20 MetS rats (red squares) for (**B**–**E**,**H**–**J**,**L**), and from N = 11 control rats and N = 14 MetS rats for K. The statistical analyses were performed by the Student’s unpaired *t*-test (**G**,**I**–**L**), Mann–Whitney test (**C**–**E**,**H**), or two-way ANOVA with repeated measures followed by Bonferroni’s post hoc test (**B**,**F**); * *p* < 0.05, *** *p* < 0.001.

To confirm the development of MetS features, morphological and biochemical parameters, as well as results of the Glucose Tolerance Test (GTT), were evaluated. Figure 1 displays the biochemical parameters of MetS rats and corresponding controls at the end of the experimental treatment. The consumption of 30% sucrose solution for four months led to obesity, evidenced by an increase to 138 ± 5% in body weight (BW) (Figure 1A,B). Importantly, according to the tibia length (TL), there was no variation in animal length (Figure 1C). From the first month of sucrose administration, MetS rats exhibited higher gain in BW, leading to a significant difference at the end of sucrose administration (control: 456.30 ± 9.08 g, MetS: 629.00 ± 22.76 g, *p* < 0.0001; Figure 1B), reflected in the BW/TL ratio (Figure 1D).

MetS rats did not show fasting hyperglycemia (Figure 1E); however, in the GTT (Figure 1F), their glycemia increased notably at 15, 30, and 60 min, which was reflected in the augmented area under the curve (Figure 1G). Moreover, MetS rats showed both fasting hypertriglyceridemia (Figure 1H) and a reduction in HDL-C levels (Figure 1I) compared to control rats, leading to a higher (~2-fold) TGL/HDL-C ratio than in the control group (Figure 1J), indicative in humans of increased cardiovascular risk [18]. MetS rats also showed an increased abdominal circumference/TL ratio (Figure 1K) compared with the control animals, as well as a greater mass of adipose tissue (mesenteric, epididymal, and retroperitoneal fat: MesFat, EpiFat, and RetroFat, respectively), resulting in a significant increase in the adiposity index (Figure 1L).

These results indicate that rats receiving 30% sucrose in drinking water for four months exhibited features consistent with MetS, fulfilling four (central obesity, hypertriglyceridemia, decreased HDL-C, and glucose intolerance) of the diagnostic criteria established for MetS in humans [1].

### 2.2. Altered Cardiac Function in MetS Rats

**Figure 2:**
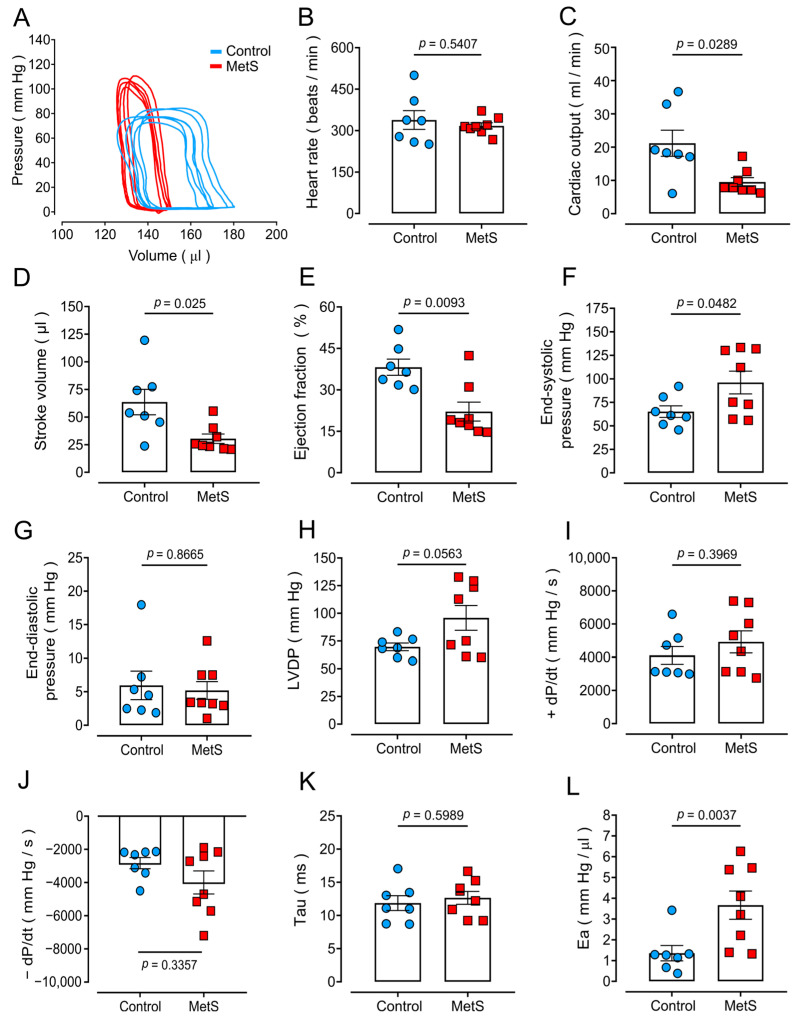
**Characterization of cardiac dysfunction in MetS rats.** Points represent individual determinations. (**A**) Representative PV loops from control (blue traces) and MetS (red traces) rats. (**B**) Heart rate. (**C**) Cardiac output. (**D**) Stroke volume. (**E**) Ejection fraction. (**F**) End-systolic pressure. (**G**) End-diastolic pressure. (**H**) Left ventricular developed pressure (LVDP). (**I**) Maximum rate of pressure change during systole (+dP/dt). (**J**) Maximum rate of pressure change during diastole (−dP/dt). (**K**) Isovolumetric relaxation constant (τ). (**L**) Arterial elastance (Ea). Data are means ± SEMs from N = 7 control rats (blue circles) and N = 8 MetS rats (red squares). Statistical analyses were performed with Student’s unpaired *t*-test.

A previous study by our laboratory [16] revealed abnormal diastolic Ca^2+^ leakage (in the form of spontaneous Ca^2+^ waves) in isolated cardiomyocytes from MetS rats, which can alter heart function and be the substrate for sympathetic-driven cardiac arrhythmias. To determine whether MetS rats exhibit cardiac dysfunction, we assessed heart activity in vivo with an open-chest approach and recorded PV loops via a conductance catheter placed into the LV. Figure 2A shows representative PV loops from control and MetS rats. The PV loops from MetS rats exhibited an altered shape with a narrower volume range and larger pressure range compared with those from control rats (Figure 2A).

HR was similar in both experimental groups (Figure 2B), but CO was markedly decreased (−55%) in the MetS group (Figure 2C) compared to control animals, attributed to a significant reduction in SV (Figure 2D) and resulting in a 43% decrease in EF (Figure 2E). MetS rats showed augmented LV systolic function in resting/baseline conditions, evidenced by higher end-systolic pressure (Figure 2F) and LVDP (Figure 2H), with no significant changes in +dP/dt (Figure 2I). LV diastolic functions remained unaltered in MetS rats, as evidenced by similar values for end-diastolic pressure (Figure 2G), dP/dt (Figure 2J), and the isovolumetric relaxation constant (τ, Figure 2K). However, Ea (Figure 2L), a measure of afterload, was significantly higher (2.7-fold) in MetS animals. Together, these findings suggest compromised cardiac performance in MetS rats.

### 2.3. Higher Susceptibility of MetS Rats to Lethal Arrhythmia Induced by β-Adrenergic Stimulation

**Figure 3:**
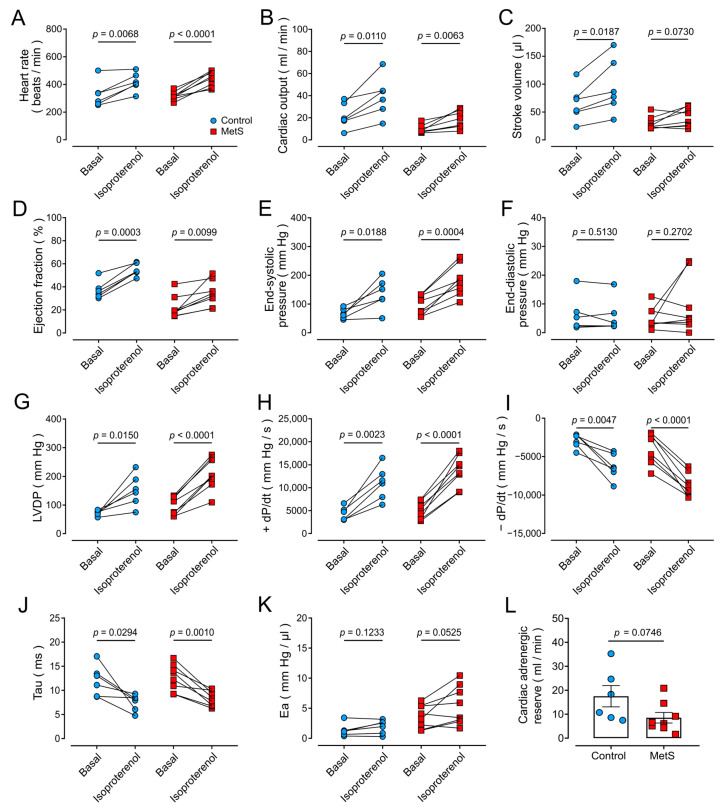
**Effect of β-adrenergic stimulation on cardiac and hemodynamic parameters.** Points represent individual determinations of N = 6 control rats (blue circles) and N = 8 MetS rats (red squares) under the basal condition and following isoproterenol stimulation. (**A**) Heart rate. (**B**) Cardiac output. (**C**) Stroke volume. (**D**) Ejection fraction. (**E**) End-systolic pressure. (**F**) End-diastolic pressure. (**G**) Left ventricular developed pressure (LVDP). (**H**) Maximum rate of pressure change during systole (+dP/dt). (**I**) Maximum rate of pressure change during diastole (−dP/dt). (**J**) Isometric relaxation constant (τ). (**K**) Arterial elastance (Ea). (**L**) Cardiac adrenergic reserve (CO_15s_–CO_Basal_). Statistical analyses were performed with Student’s paired (**A**–**K**) or unpaired (**L**) *t*-test.

To evaluate the β-adrenergic response, the non-selective β-adrenergic agonist isoproterenol (300 µg/kg) was directly administered into the LV. Doses were adjusted to the significantly higher BW and HW of MetS rats (see Table 1). Figure 3 shows parameters of cardiac function before and after (15 s) isoproterenol administration. As expected, in the control group, isoproterenol produced positive inotropic and chronotropic responses, as evidenced by the increase in heart rate, CO, SV, and EF (Figure 3A–D), as well as contraction (end-systolic pressure, LVDP, and +dP/dt; Figure 3E,G,H) and relaxation (−dP/dt and τ, Figure 3I–J) parameters.

In MetS rats, isoproterenol induced a significant increase in HR (Figure 3A), CO (Figure 3B), EF (Figure 3D), end-systolic pressure (Figure 3E), LVDP (Figure 3G), +dP/dt (Figure 3H), −dP/dt (Figure 3I), and τ (Figure 3J), with no significant changes in SV (Figure 3C), end-diastolic pressure (Figure 3F), or Ea (Figure 3K). The β-adrenergic cardiac reserve (Figure 3L), measured as the difference between the maximum CO value after 15 s of isoproterenol administration and basal CO (CO_15s_–CO_Basal_), was reduced by ~50% in MetS rats, although the comparison with values from control animals did not yield statistical significance (*p* = 0.0730). To further analyze whether the effect of isoproterenol differed from that in control rats, we calculated the difference between measurements before and after isoproterenol administration for each cardiac hemodynamic parameter. Our data showed no significant differences between groups (Table 2), indicating that the β-adrenergic response is similar in MetS and control animals.

**Figure 4:**
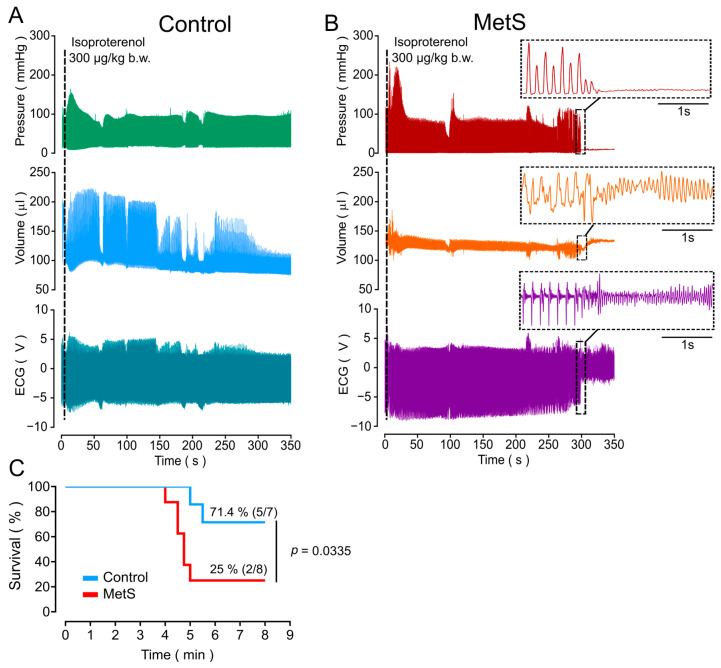
**Isoproterenol-induced lethal arrhythmia in MetS rats.** Representative recordings of LV pressure (mm Hg), volume (µL), and ECG (V) for control ((**A**), N = 7 rats) and MetS ((**B**), N = 8) rats, before and following isoproterenol administration, indicated by the dotted line. The inserts in MetS recordings are amplifications showing the presence of asystole and the appearance of arrhythmia resembling ventricular fibrillation after ~5 min of β-adrenergic stimulation. (**C**) Survival analysis was performed with the Mantel–Cox test.

After ~5 min, isoproterenol administration induced lethal arrhythmia in six of eight MetS rats (75%) and two of seven control animals (28.6%). Figure 4 shows representative raw data of pressure, volume, and ECG recorded with a conductance catheter during cardiac function recordings for control (Figure 4A) and MetS (Figure 4B) rats. During arrhythmia, MetS rats exhibited an ECG pattern that resembles ventricular fibrillation (see inserted ECG image). The Mantel–Cox survival analysis (Figure 4C) revealed a significant difference between the groups (*p* = 0.0335), with a survival rate of 71.4% (5/7) in control rats and 25% (2/8) in MetS rats, indicating increased lethality associated with the MetS condition.

To calculate the relative risk (RR) of lethal arrhythmia after β-adrenergic stimulation in the MetS rats, we performed Fisher’s exact test, with RR = 2.625 (95% confidence interval: 0.923, 9.499), indicating that the MetS rats had an increased (~2.6-fold) risk of presenting the event (arrhythmia and subsequent death) compared to controls, although the *p* value was not statistically significant (*p* = 0.1319).

### 2.4. Ultrastructural Cardiac Remodeling in MetS Hearts

To evaluate cardiac remodeling, heart structures were weighed and the corresponding ratios were calculated (Table 1) as an indirect measure of hypertrophy [19].

HW increased in MetS rats compared to the control animals, but RVW and LVW were not different. Due to the variability in body mass for both experimental groups, relationships such as HW/BW, LV/HW, and RW/HW were not reliable parameters with which to determine cardiac remodeling. Therefore, we calculated ratios over the tibia length (TL), a stable metric with no difference between groups at the conclusion of the experimental treatment (Figure 1L). This approach allowed for the evaluation of cardiac hypertrophy according to a previous report [19]. TL is independent of age-related changes and represents a more stable reference parameter in experimental protocols where body weight may vary [20]. The HW/TL ratio increased in a significant manner in MetS rats (*p* = 0.0089), whereas a non-significant increase (*p* = 0.0870) was observed for the LVW/TL ratio (Table 1).

**Figure 5:**
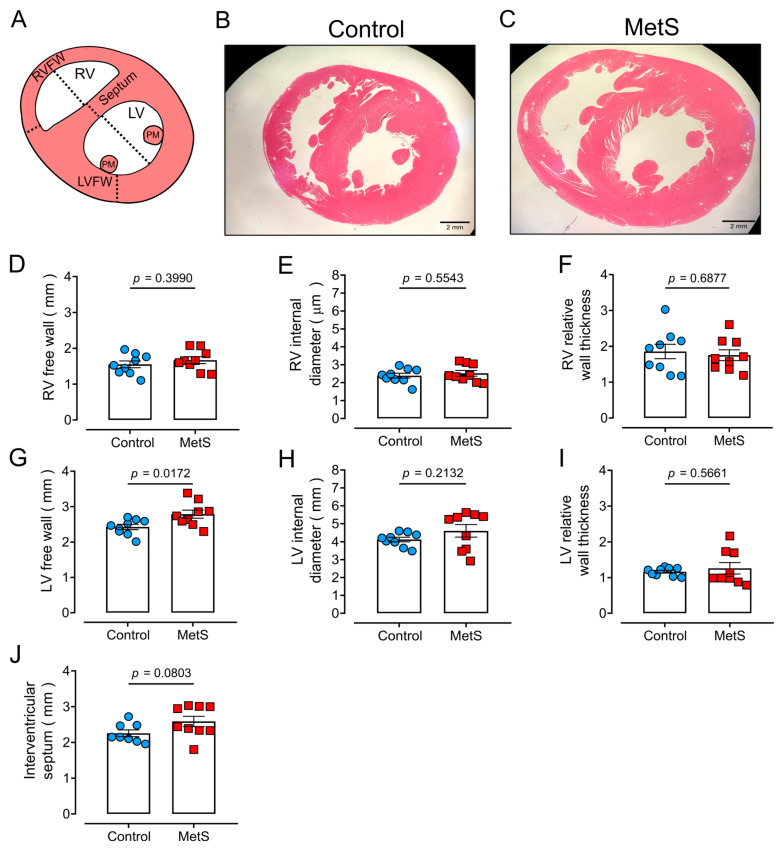
**Cardiac macroscopic parameters from heart H&E staining.** Data points represent individual determinations. (**A**) Schematic representation of the heart illustrating the parameters analyzed. Representative images of hearts from control (**B**) and MetS (**C**) rats. Right ventricle (RV): (**D**) free wall (RVFW), (**E**) internal diameter, and (**F**) relative wall thickness. Left ventricle (LV): (**G**) free wall (LVFW), (**H**) internal diameter, and (**I**) relative wall thickness. (**J**) Thickness of the interventricular septum. Data are means ± SEMs from N = 9 animals per group. Statistical analyses were performed with the Student’s unpaired *t*-test. PM: papillary muscle.

To further evaluate cardiac remodeling, both wall thickness and the internal diameter were measured using hematoxylin–eosin (H&E) staining on 5 µm thick cross-sectional slices of both heart ventricles, as illustrated by Figure 5A. Figure 5 (panels B and C) shows representative images of the hearts from control (Figure 5B) and MetS (Figure 5C) animals. The thickness of free walls and the internal diameters of both ventricles were determined to calculate relative wall thickness, an indicator of hypertrophy, as the sum of the interventricular septum and the wall thickness (RV or LV) divided by the internal diameter of the corresponding ventricle [21]. For RV, there were no changes in the thickness of the free wall (Figure 5D) or the internal diameter (Figure 5E), resulting in a lack of difference in the relative wall thickness (Figure 5F). Likewise, there was no statistical difference in septum thickness (Figure 5J). The LV free wall thickness was significantly larger in the MetS animals (Figure 5G, *p* = 0.0172), with no significant difference in either the LV internal diameter (Figure 5H) or relative wall thickness (Figure 5I). These findings suggest that cardiac remodeling in MetS rats likely occurs in a region-specific manner, predominantly in the LV free wall, while the RV and septum remain structurally unaffected and do not reflect hypertrophy.

Cardiac fibrosis was evaluated with Masson’s trichrome staining (Figure S1A) to measure collagen deposition. There was no significant difference in the percentage of collagen deposition between control and MetS rats (control: 1.45 ± 0.31%, MetS: 1.97 ± 0.36%; Figure S1B). Heart ultrastructure was further examined by transmission electron microscopy in three animals per experimental group (Figure S1C,D). Figure S1E shows a trend toward a higher number of mitochondria (*p* = 0.0515) in MetS rats, while the mitochondrial area remained similar between groups (Figure S1F). These results suggest that, despite the absence of overt cardiac fibrosis, MetS hearts may undergo subtle ultrastructural changes.

### 2.5. Hyperadrenergic Basal State in MetS Rats

**Figure 6:**
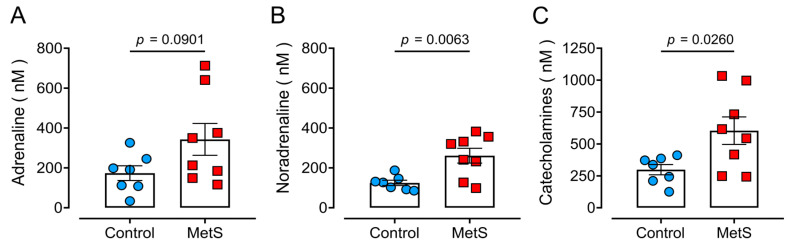
**Serum catecholamine levels in MetS rats.** (**A**) Adrenaline, (**B**) noradrenaline, and (**C**) total catecholamines. Each dot represents the average of duplicate determinations from a single animal. Data are means ± SEMs from N = 7 (control, blue circles) or N = 8 (MetS, red squares) animals. The statistical analysis was performed with the Student’s unpaired *t*-test.

MetS increases SNS activity [3]. Therefore, we aimed to determine whether MetS rats showed augmented SNS activity and/or abnormal fight-or-flight responses. Serum adrenaline levels were similar in MetS rats (control: 173.4 ± 38.7 nM, MetS: 349.8 ± 83.7 nM; Figure 6A). However, noradrenaline levels were higher in the MetS animals (control: 120.4 ± 13.5 nM, MetS: 288.4 ± 45.8 nM; Figure 6B), with the difference being statistically significant (*p* = 0.0063). The arithmetic sum of adrenaline plus noradrenaline (total catecholamine levels) resulted in a statistically significant increase in MetS rats (*p* = 0.0260, Figure 6C). These values indicate that MetS leads to a chronic elevation in sympathetic activity, similar to that observed in humans.

### 2.6. Altered βAR Expression in MetS Hearts

**Figure 7:**
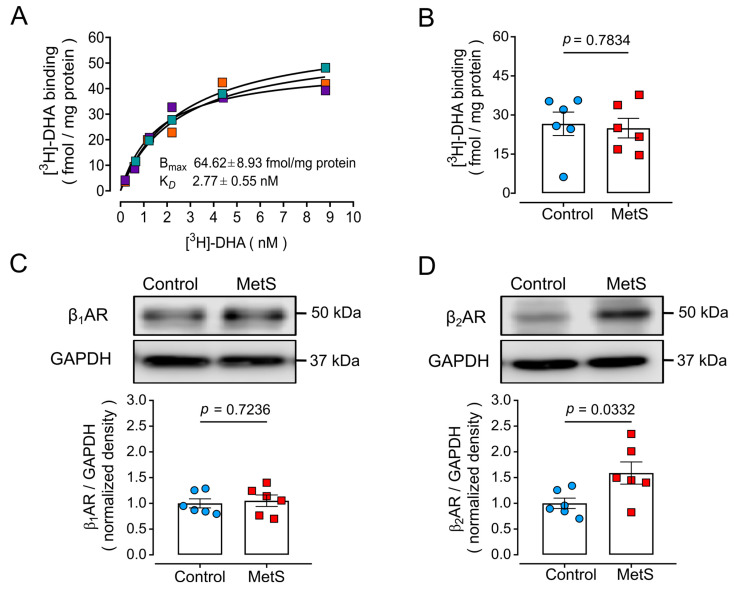
**Total βAR and protein expression of β_1_AR and β_2_AR in LV homogenates of control and MetS rats**. (**A**) Saturation curve of specific [^3^H]-dihydroalprenolol ([^3^H]-DHA) binding to LV membranes from three rats outside the experimental groups. The symbols represent the average of triplicates for individual determinations indicated by different colors. The lines correspond to the best fit of a hyperbola. (**B**) Specific [^3^H]-DHA binding from one-point binding assays for LV membranes (N = 6 animals per group). Each dot represents the average of quadruplicate determinations. (**C**,**D**) Immunodetection of β_1_ARs and β_2_ARs. Representative Western blots and analyses for β_1_AR (**C**) or β_2_AR (**D**) in LV extracts for N = 6 animals per group. Statistical analyses were performed with the Student’s unpaired *t*-test.

Augmented serum catecholamine levels could affect βAR expression by inducing receptor desensitization and/or downregulation to counteract basal hyperstimulation by adrenaline and noradrenaline. Therefore, we analyzed the protein expression of βARs in LV cell membranes. Saturation [^3^H]-dihydroalprenolol ([^3^H]-DHA) binding in three LV membrane preparations from rats outside the experimental groups (Figure 7A) yielded a maximum specific binding (B_Max_) of 64.62 ± 8.93 fmol/mg protein and a constant dissociation (K_D_) of 2.77 ± 0.55 nM, values similar to those previously reported [22]. In one-point radioligand binding assays (Figure 7B), there was no significant difference in [^3^H]-DHA binding (26.61 ± 4.49 and 24.95 ± 3.76 fmol/mg protein for control and MetS rats, respectively; *p* = 0.7834). [^3^H]-DHA is a non-selective antagonist at βARs, and these results indicate that βAR total density does not change despite the hyperadrenergic state developed in MetS rats.

We evaluated βAR protein expression in LV protein extracts by Western blotting with selective antibodies against β_1_ARs (Figure 7C) or β_2_ARs (Figure 7D). The observed molecular mass for both β_1_AR and β_2_AR was ~50 kDa, as reported by the antibody supplier. Whereas β_1_AR levels remained unchanged in the MetS group compared to the control group, β_2_AR expression increased significantly (~1.5 fold) in LV extracts of MetS rats.

### 2.7. Altered Expression of Proteins of the β-Adrenergic Signaling Pathway in LV Extracts of MetS Rats

**Figure 8:**
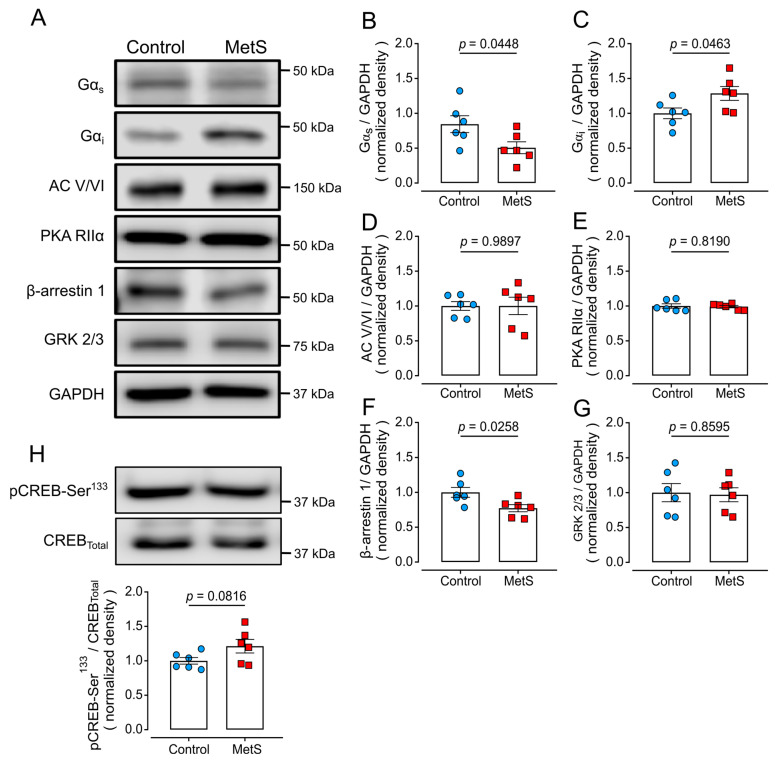
**Expression of βAR signaling proteins in LV extracts of MetS hearts**. Points represent individual determinations. Representative Western blots (**A**) and the quantification of proteins involved in βAR signaling: (**B**) Gα_s_; (**C**) Gα_i_; (**D**) adenylyl cyclase (AC) V/VI; (**E**) protein kinase A (PKA) regulatory subunit RIIα; (**F**) β-arrestin 1; and (**G**) G-protein coupled receptor kinase 2/3 (GRK-2/3). (**H**) Representative Western blots of pCREB-Ser^133^ and total CREB (upper images) and the corresponding quantitative analysis (lower graph). Data are means ± SEMs from N = 6 animals per group. Statistical analyses were performed with the Student’s unpaired *t*-test.

We further evaluated alterations in the β-adrenergic signaling pathway (Figure 8) by determining the protein levels of several proteins that participate in this pathway (Figure 8A). β_1_ARs and β_2_ARs couple to Gα_s_ proteins and activate adenylyl cyclase (AC) to induce cAMP formation [23], although β_2_ARs can also couple to Gα_i/o_ proteins that inhibit AC activity [12]. In MetS heart homogenates we found a significant decrease in Gα_s_ protein levels (*p* = 0.0448, Figure 8B) and a significant increase in Gα_i_ proteins (*p* = 0.0463, Figure 8C). AC isoforms V and VI are predominantly expressed in the heart [23], and no significant changes were detected for their expression (predicted molecular mass: 120–150 kDa, Figure 8D). The second messenger cAMP binds to the PKA regulatory subunits, with the RIIα isoform being the most prevalent in the heart [24] and preferentially activated by βARs [25]. The PKA-RIIα molecular mass is 55 kDa, and we did not detect changes in MetS rats compared to control animals (Figure 8E).

Upon exposure to agonists, βARs experience homologous desensitization triggered by GRK-mediated receptor phosphorylation, which in turn leads to the recruitment of β-arrestins and βAR internalization. We found decreased β-arrestin-1 protein levels (*p* = 0.0258, Figure 8F) but no changes in GRK-2/3 levels in MetS rats (Figure 8G). Finally, we determined CREB phosphorylation at Ser^133^ (pCREB-Ser^133^) as an indirect measure of constitutive PKA activity, but no statistical difference was detected (Figure 8H).

## 3. Discussion

**Figure 9:**
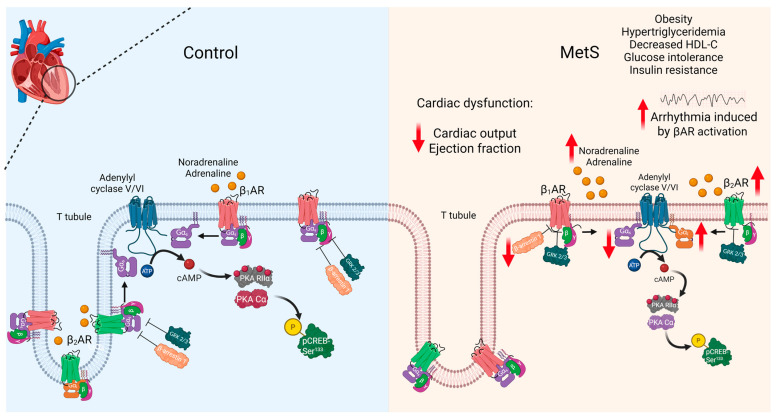
**Altered β-adrenergic system in MetS hearts.** MetS rats presented cardiac dysfunction, evidenced by a reduction in cardiac output and ejection fraction. MetS rats also had a higher incidence of lethal arrhythmia induced by βAR activation with isoproterenol. The left ventricle of MetS rats showed decreased levels of Gα_s_ proteins and β-arrestin 1, but increased levels of β_2_ARs and Gα_i_ proteins. ↑, increase; ↓, decrease. Created in BioRender.com.

The main findings of this study are that MetS leads to pronounced obesity, cardiac dysfunction, and the altered expression of proteins related to the βAR signaling pathway, which could therefore predispose individuals to lethal arrhythmia induced by β-adrenergic stimulation (Figure 9).

We showed that the chronic (four months) consumption of sugar (30% in drinking water) leads to the development of features similar to those present in humans diagnosed with MetS. Our experimental model of MetS in rats developed insulin resistance (indirectly determined by the GTT) and dyslipidemia, included in the diagnostic criteria for MetS in humans. Specifically, MetS rats exhibit pronounced obesity characterized by the marked accumulation of adipose tissue, a key factor in the pathophysiology of MetS [26]. Abdominal obesity is related to insulin resistance, and previous work from our laboratory showed that MetS rats developed hyperinsulinemia and impaired insulin-induced glucose uptake by isolated cardiomyocytes [15], a finding corroborated in cardiomyocytes from MetS patients [27].

There exist experimental protocols to induce MetS by combining a high-fat diet with the systemic administration of streptozotocin, a drug widely used to produce type 1 or type 2 diabetes mellitus by destroying pancreatic islet β-cells [28]. However, streptozotocin also affects the peripheral and central nervous systems [29], introducing an inevitable variable that per se can alter the cardiac and nervous systems. Therefore, we consider that the experimental model based on sugar consumption offers advantages for replicating MetS features compared with other experimental models (reviewed in reference [30]).

We previously evidenced abnormal Ca^2+^ leakage in MetS cardiomyocytes in the form of spontaneous Ca^2+^ waves exacerbated after β-adrenergic stimulation with isoproterenol [16], and this study sought to provide insight into how this phenomenon impacts both the global function and the β-adrenergic response of the heart. We report here that MetS rats present an ~40% reduction in EF compared to control animals, which is attributed to a reduced stroke volume, likely caused by increased arterial elastance, implying a greater afterload to be overcome by the heart, and potentially related to obesity. These results are in agreement with our findings of the decreased amplitude of electrically induced Ca^2+^ transients and reduced contractility in MetS isolated cardiomyocytes [31], implying systolic dysfunction. Despite a lack of effect on diastolic parameters, such as end-diastolic pressure, −dP/dt, or τ, we cannot discard diastolic dysfunction because MetS cardiomyocytes present diastolic Ca^2+^ leakage [16]. Altogether, our results show impaired cardiac function in MetS rats, which might contribute to the higher risk of heart failure in patients with MetS or insulin resistance [32,33].

We evaluated distinct factors that could contribute to cardiac dysfunction. First, although HW and the HW/TL ratio, indicators of hypertrophy [19], were greater for MetS rats, there were no significant differences in LV, RV, the LV/TL and RV/TL ratios, and relative RV or LV wall thickness, leading to a lack of evidence for heart hypertrophy at the structural level. However, we did not measure serum markers of hypertrophy [34], such as atrial natriuretic peptide, type b natriuretic peptide, or troponin I, that would have provided further evidence. We cannot attribute cardiac dysfunction to fibrosis according to our findings; further studies are required to evaluate the development of fibrosis at later stages.

Regarding the analysis of cardiac ultrastructure by transmission electron microscopy, the lack of a relaxant before sample processing for microscopy [35] did not allow for full heart relaxation, thus preventing the measurement of sarcomere length under constant conditions. However, we observed a trend toward increased mitochondria number in the hearts of MetS rats, which may represent a compensatory response to metabolic or functional demands. In this regard, previous studies have shown that mice fed a fructose-rich diet for three weeks exhibit a decreased mitochondrial area [36], suggesting that metabolic alterations can impact mitochondrial structure and compromise mitochondrial function. Hearts from rats with hypertriglyceridemia induced by sugar consumption have decreased activity of the pyruvate dehydrogenase complex (located in the inner mitochondrial membrane) and reduced ATP levels, along with altered cardiac performance [37], supporting the idea that the impairment of mitochondrial dynamics contributes to myocardial damage and cardiac dysfunction [38,39].

Regarding cardiac function, systolic dysfunction induced by MetS could involve abnormal β-adrenergic responses. MetS rats have increased levels of serum catecholamines that could be explained by the enhanced spillover of adrenaline and noradrenaline from sympathetic fibers, as occurs in MetS patients [3,5,6] and diabetic animals [40]. This phenomenon would imply βAR hyperactivation and desensitization, leading to receptor downregulation.

Radioligand binding experiments showed no difference in total βAR density in LV membranes; levels of 25–27 fmol/mg protein are within the range previously reported for βARs in rat cardiomyocytes (16–40 fmol/mg protein; [41]). However, via Western blotting, we detected increased β_2_AR density (150%). In rats, β_1_ARs compose ~75% of total βARs, while β_2_ARs represent ~25% in LV [9], and the increase in β_2_AR number could therefore not suffice to significantly modify total heart βAR density. β_2_AR expression increases in some pathologies, such as heart failure [42], and its function is also modified in insulin resistance conditions [14,43].

We found increased expression of Gα_i_ proteins with decreased expression of β-arrestins in MetS hearts. The phosphorylation of β_2_ARs by GRK-2 and PKA in the desensitization process favors receptor coupling to Gα_i/o_ proteins [14,44] as a protective mechanism in heart failure, and the decrease in β-arrestin 1 could reduce β_2_AR desensitization as a counteracting mechanism [45]. We also detected decreased Gα_s_ protein levels, which, along with the increase in Gα_i_ proteins, would lead to altered βAR signaling. In rat cardiomyocytes, Gα_s_ protein levels are relatively in excess compared to βARs and ACs. Each cardiomyocyte contains ~2.1×10^5^ βARs and 4.7 × 10^7^ Gα_s_ molecules, but only 6 × 10^5^ Gα_s_-AC complexes form in response to maximal Gα_s_ activation [46]. Therefore, it is possible that the decrease in Gα_s_ levels does not impact the global β-adrenergic response due to the excess of Gα_s_ proteins. In fact, MetS rats responded to βAR activation with positive chronotropic, inotropic, and lusitropic responses during the first seconds of activation, as indicated by the increase in heart rate, CO, EF, end-systolic pressure, LVDP, and +dP/dt, and reduced −dP/dt as well as τ after isoproterenol administration, despite cardiac performance being compromised in resting conditions.

At the molecular level, alterations in βAR signaling were evident and consistent with our previous results [16]. In fact, the phosphorylation of RyR at Ser^2808^ and PLN at Ser^16^ does not increase in response to βAR stimulation in MetS rats. In contrast, the phosphorylation at Ser^2814^ of RyR is significantly enhanced, and the Thr^17^ of PLN exhibits elevated basal phosphorylation. These modifications were associated with an increased frequency of Ca^2+^ sparks upon βAR activation. Together, these results support the idea that βAR signaling is disrupted in the hearts of MetS rats at levels both upstream and downstream of the pathway.

A relevant finding of this study was the higher incidence of lethal arrhythmia in MetS rats induced by βAR stimulation by isoproterenol. This response could be related to our previous results [16], showing that, in cardiomyocytes from MetS rats, spontaneous Ca^2+^ waves are exacerbated by β-adrenergic stimulation along with Ca^2+^ sparks, effects that can lead to ventricular arrhythmias [47,48]. Similarly, in another rat MetS model, β-adrenergic stimulation increased the incidence of both premature ventricular contractions and ventricular fibrillation [49]. This effect was attributed to an inflammatory state originating from elevated serum levels of interleukin 1β (IL-1β), tumoral necrosis factor α (TNF-α), and interleukin 6 (IL-6). These experiments were performed with an isolated heart perfused with isoproterenol (10 nM), and their outcomes are consistent with our results. The incidence of ventricular fibrillation (73%) was similar to that reported in this study (71.4%).

The pro-arrhythmic effect of β-adrenergic stimulation could be explained by increasing L-type Ca^2+^ channel activity, which would result in intracellular Ca^2+^ mishandling that alters the cardiac conduction system and prolongs the repolarization phase of the action potential. Both conduction abnormalities create a substrate for re-entry circuits and triggered activity, thus facilitating the development of malignant arrhythmias, particularly in pathological conditions like MetS [50]. It should be mentioned that under our conditions rats were subjected to exacerbated adrenergic stimulation because the concentration of isoproterenol in the solution injected into LV was very high (14.3 mM), although it rapidly diluted into the ventricular blood volume (~180 µL end-diastolic volume for control rats).

Our experimental design involved in vivo responses, with both the sympathetic and parasympathetic nervous systems modulating cardiac performance. Consequently, our study provides an assessment that devotes more attention to the pathophysiological conditions.

### Limitations of This Study

We acknowledge several limitations of our study. Firstly, cardiac hemodynamic parameters observed in control animals, such as SV and EF, were comparatively lower than those reported in previous studies [51]. This discrepancy is attributable to the deep level of anesthesia and the reduction in intrathoracic pressure associated with the open-chest procedure, both of which can compromise cardiac performance measurements.

The potential mechanisms of arrhythmia induced by βAR activation were not explored. Further investigation should include detailed electrophysiological analyses to assess the effects of βAR stimulation on the electrical activity of key cardiac structures, mainly the sinoatrial node. This would provide a more comprehensive understanding of how β-adrenergic stimulation influences cardiac rhythm and the susceptibility to arrhythmias in the context of MetS.

Moreover, binding determinations and immunodetection assays were performed on the whole cardiac (LV) tissue, and at least for humans the LV is composed of 50% cardiomyocytes, whereas the remaining 50% of cells correspond to other types, such as endothelial cells, immune cells, smooth muscle cells, and neuronal cells, among others [52]. It would therefore be necessary to evaluate the β-adrenergic system in cardiomyocytes from MetS rats to assess the relative contribution of other cells to the parameters evaluated. Furthermore, α_1B_-ARs are also expressed in the heart and would participate in the functional responses to adrenaline and noradrenaline [53], and this condition requires further evaluation, such as by the pharmacological blockade of α_1_-adrenoceptors when evaluating responses to the endogenous agonists.

Secondly, a major limitation for the [^3^H]-DHA binding assays is the lack of discrimination between β_1_ARs and β_2_ARs because the radioligand is a non-selective βAR antagonist. We addressed partially this issue by measuring by Western blotting the expression of β_1_ARs and β_2_ARs; however, the resolution depends critically on the antibody affinity.

Thirdly, this work did not evaluate βAR post-translational modifications that play relevant roles in receptor function, for example, O-glycosylation, proteolytic cleavage, and oxidative stress for β_1_ARs that preferentially activate the cAMP- or mitogen-activated protein kinase signaling pathways [54,55,56], or receptor phosphorylation by GRKs, PKA, or protein kinase C that results in β_1_AR and β_2_AR desensitization [14].

Fourthly, we did not address the probable causes of more abundant mitochondria in MetS hearts. Further research is warranted to define the mechanisms of cardiac dysfunction, to elucidate at the cellular and molecular levels the implications of altered βAR signaling pathways, and to decipher the mechanisms underlying the increased incidence of lethal arrhythmia in this experimental MetS model.

Finally, we acknowledge the importance of addressing sex-related differences in MetS pathophysiology, which represents a limitation of the present study. This work follows previous studies conducted on male rats [15,16], as we have observed that four months of sucrose consumption in female rats results in a smaller increase in TGs and no reduction in HDL-C, the latter being one of the criteria for MetS used in this study. Therefore, the further characterization of MetS models in female animals is required to explore potential sex-specific outcomes regarding β-adrenergic signaling and arrhythmia susceptibility.

## 4. Materials and Methods

The animals were provided by the vivarium of Cinvestav, and all the experimental procedures were in accordance with the ethical guidelines for the care and use of laboratory animals (Mexican Norm NOM-062-ZOO-1999 and National Institutes of Health Guide for the Care and Use of Laboratory Animals updated in 2011), under the protocol 0105-14, approved by CICUAL (Comité Interno para el Cuidado y Uso de los Animales de Laboratorio, Cinvestav).

### 4.1. Sucrose-Induced MetS in Rats

Male Wistar rats (21 postnatal days) were randomly distributed into two groups (control and MetS) and were maintained under standard conditions (12 h dark–light cycle and room temperature of 22 ± 2 °C). The control group received plain water, while the MetS group received 30% sucrose (refined commercial sugar) in the drinking water. Both groups were fed ad libitum with standard rat chow (PicoLab Rodent Diet 20 LabDiet; St. Louis, MO, USA) for four months, as reported previously [15,16], with the monthly monitoring of body weight (BW).

### 4.2. Biochemical Characterization of the MetS Model

At the end of the MetS induction period, rats were kept under 12 h of fasting to measure biochemical parameters. Glucose was determined in fresh blood samples with a conventional glucometer (Accu-Chek; Roche, Basel, Switzerland). Triglycerides (TGs) and high-density lipoprotein cholesterol (HDL-C) were measured with lipid panel strips (PTS lipid panel) and a CardioCheck PA analyzer (PTS Diagnostics, Indianapolis, IN, USA).

### 4.3. Determination of Obesity

Animals were anesthetized with an intraperitoneal injection of sodium pentobarbital (65 mg/kg; Laboratorios Pisa, Guadalajara, Jalisco, Mexico), and injected with heparin (1000 U/kg, i.p.; Laboratorios Pisa) to prevent blood coagulation. Under anesthesia, the abdominal circumference was determined at the iliac crest level with a measuring tape. A thoracotomy was performed to excise the heart, and the organ was then allocated for the experimental procedures described below. Thereafter, the fat tissue (MesFat, EpiFat, and RetroFat) was carefully dissected and weighed to calculate the adiposity index (AI) as an indicator of obesity magnitude according to the following formula [18]:

AI = (MesFat + EpiFat + RetroFat)/(Body weight (BW) × 100(1)

### 4.4. Glucose Tolerance Test (GTT)

The GTT, an indirect measure of insulin resistance, was performed by the intraperitoneal injection of a 50% glucose solution (2 g/kg; 4 mL/kg) and the determination of glycemia in a blood sample obtained from a tail vein at 0, 15, 30, 60, 90, and 120 min with a conventional glucometer (Accu-Chek). Values were plotted as a function of time, and the area under the curve was computed [15].

### 4.5. Determination of Serum Catecholamine Levels

The serum was separated by centrifugation (2000× *g*, 10 min) from blood samples obtained during heart dissection. Non-hemolyzed serum samples were used to measure adrenaline and noradrenaline levels by an ELISA (Abnova kit, KA1887; Taipei City, Taiwan) according to the protocol provided by the manufacturer. The absorbance was measured at 450 nm using an Epoch microplate reader (Bio Tek, Winooski, VT, USA). The concentrations of adrenaline and noradrenaline (nM) were interpolated from a standard curve fitted to an Akima spline.

### 4.6. Evaluation of Heart Hypertrophy and Fibrosis

To evaluate hypertrophy, wet weight was determined for the heart (HW), the left ventricle (LVW, left ventricle free wall along with interventricular septum), and the right ventricle free wall (RVW). Nine hearts per experimental group were used to obtain tissue slices at the middle ventricular level, and the slices were processed for hematoxylin–eosin (H&E) staining [21]. Photographs were taken with a stereo microscope (SMZ800N; Nikon, Japan) previously calibrated (4.36 μm/pixels), and the posterior side of the right ventricle free wall (RVFW), left ventricle free wall (LVFW), interventricular septum (IS), and internal diameters of both the LV and right ventricle (RV) were measured from the images using NIS-Elements software (Nikon, Japan). Masson trichrome staining was also performed on independent slices of the same hearts to analyze collagen deposition in three random sections of LVFW per animal acquired with the same stereo microscope and analyzed with Fiji software (ImageJ v1.54f; NIH, Bethesda, MD, USA).

### 4.7. Evaluation of Cardiac Function

After anesthesia with pentobarbital (65 mg/kg, i.p.) and proper body temperature control, a tracheostomy was performed, and the animals were intubated and connected to a mechanical ventilator at 70 breaths/min. The heart was exposed with a thoracotomy in order to place an intra-cardiac conductance catheter (SPR-869 Millar; Houston, TX, USA) into the LV, behind the left anterior descendent coronary artery, to record pressure and volume with LabChart Pro software (v. 8.1.28; ADInstruments Inc., Colorado Springs, CO, USA). After stabilization, the following hemodynamic parameters were measured in basal conditions: heart rate (HR), cardiac output (CO), stroke volume (SV), and ejection fraction (EF). LV systolic function was evaluated via end-systolic pressure (ESP), left ventricular developed pressure (LVDP), maximum rate of pressure rise during systole (+dP/dt), and arterial elastance (Ea). LV diastolic function was evaluated by end-diastolic pressure (EDP), the maximum rate of pressure fall during diastole (−dP/dt), and the isovolumetric relaxation constant (τ). PV loops were also recorded. To elicit the β-adrenergic response, the non-selective β-adrenergic agonist isoproterenol (300 µg/kg; 3 µg/µL solution) was administered directly into the LV, and the parameters indicated above were evaluated. An electrocardiogram (ECG) was recorded using the same conductance catheter inserted into the LV for simultaneous pressure–volume measurements during open-chest experiments. This approach provides unipolar intraventricular ECGs, primarily used to monitor heart rate and detect the onset of arrhythmia following isoproterenol administration.

### 4.8. Preparation of LV Homogenates

Tissues were processed as previously reported [16]. Briefly, the heart was quickly excised, immersed in ice-cold Tyrode solution (NaCl 130 mM, KCl 5.4 mM, MgCl_2_ 0.5 mM, NaH_2_PO_4_ 0.4 mM, D-glucose 22 mM, and HEPES 25 mM; pH 7.4 with NaOH and pre-oxygenated with carbogen), cannulated via the aorta, and retrogradely perfused with pre-warmed (37 °C) Tyrode solution for 5 min using a Langendorff system to eliminate any residual blood. Subsequently, the LV was isolated and frozen in liquid N_2_ before pulverization. The resulting powder was homogenized three times using a Potter-Elvehjem glass homogenizer (400 rpm for 45 s) in an ice-cold lysis buffer (20 mM HEPES, 0.3 mM sucrose, and 20 mM NaF; pH 7.2 with KOH) supplemented with protease inhibitors (12 µM leupeptin, 100 µM PMSF, 500 µM benzamidine, and 153 nM aprotinin). Homogenates were centrifuged at 2000× *g* (10 min, 4 °C), and the supernatants were centrifuged again under the same conditions for 30 min. The resulting supernatants were aliquoted and stored at −70 °C until use. Protein content was quantified by the bicinchoninic acid (BCA) method.

### 4.9. Preparation of Membranes for Radioligand Binding Assays

After 5 min of perfusion of the heart with Tyrode solution, the LV was dissected and frozen in liquid N_2_. Samples were processed as previously described [22], with some modifications. Briefly, the LV was homogenized (450 rpm, 45 s, three times) in an ice-cold lysis buffer (Tris-HCl 5 mM and EDTA 5 mM, pH 7), the homogenates were centrifuged at 800× *g* for 10 min at 4 °C, and the supernatants were collected and centrifuged at 32,000× *g* for 25 min (4 °C). The pellets (cell membranes) were resuspended in an incubation buffer (Tris-HCl 75 mM and EDTA 2 mM, pH 7.5), and protein content was quantified with the BCA method.

### 4.10. [^3^H]-Dihydroalprenolol ([^3^H]-DHA) Binding Assay

For saturation analysis, membranes (150–300 µg of protein/sample) were incubated in a 100 μL volume with increasing [^3^H]-DHA concentrations (0.2–8.8 nM, specific activity: 97.9 Ci/mmol; New England Nuclear, Boston, MA, USA) diluted in an incubation buffer (Tris-HCl 75 mM and EDTA 2.5 mM, pH 7.4) in the presence and absence of alprenolol (10 µM) to define non-specific binding [57]. Equilibration was for 1 h at 30 °C and terminated by filtration through Whatman GF/B glass microfiber filters, pre-soaked in 0.3% polyethylenimine for 2 h, using a cell harvester (Brandel, Gaithersburg, MD, USA). The filters were washed three times with ice-cold buffer (Tris-HCl 25 mM, pH 7.4) and then transferred to vials containing 3.5 mL of scintillator and allowed to stand at room temperature for at least 4 h before the determination of the tritium content. Specific binding was computed as the difference between total binding and non-specific binding and expressed as fmol/mg protein. Curves were individually fitted to a one-site-specific binding equation to obtain values for the dissociation constant (K_D_) and maximum binding (B_Max_).

For single-point binding determinations, membranes (300 µg protein/sample) were incubated with ~4.5 nM [^3^H]-DHA for 1 h at 30 °C, and the reaction was terminated as described above.

### 4.11. SDS-PAGE and Western Blotting

LV extracts (Section 4.8.) were mixed with Laemmli buffer and incubated at room temperature for 5 min. Acrylamide discontinuous gradient gels (4, 8, 12, and 16%) were loaded with LV extracts (10 µg protein), and proteins were separated in denaturing conditions and transferred (4 °C, 2 h) to PVDF membranes, which were subsequently blocked at room temperature for 1 h with 5% low-fat dry milk in TBS-T buffer.

PVDF membranes were incubated overnight at 4 °C with the corresponding primary antibodies (Table S1). After 3 washes with TBS-T buffer, PVDF membranes were incubated with the corresponding secondary conjugated HRP antibody (Table S1) at room temperature for 1 h. Proteins were visualized by chemiluminescent reaction (SuperSignal West Pico Chemiluminescent Substrate; Thermo Fisher Scientific, Waltham MA, USA), and the relative amount of protein was determined via densitometric analysis using FIJI software (v1.54f; NIH, Bethesda, MD, USA).

### 4.12. Statistical Analysis

All experimental data are presented as the means ± standard errors (SEMs) and the number of animals (N) for each experiment is indicated in each figure legend. Data were tested for normality with the Shapiro–Wilk test, and a two-tailed Student’s *t*-test was performed in the case of a normal distribution; otherwise, the Mann–Whitney U test was conducted. Repeated-measures two-way ANOVA was performed to analyze the GGT and the time course of weight increase, followed by a Bonferroni post hoc analysis. Cardiac hemodynamic parameters after isoproterenol administration were analyzed with a Student’s paired *t*-test. The standard curves for adrenaline and noradrenaline were fitted to an Akima spline, and sample values were interpolated from the curves. Survival curves were compared by performing the Mantel–Cox test. Fisher’s exact test was performed to calculate the relative risk (RR) for arrhythmia and subsequent death in MetS rats. *p* values < 0.05 were considered statistically significant. All analyses were performed using Prism v. 8 (Graph Pad Software, San Diego, CA, USA).

## 5. Conclusions

MetS rats develop cardiac dysfunction evidenced by a significant decrease in CO, EF, and SV, presumably related to mitochondrial damage in the absence of evident hypertrophy and fibrosis. Moreover, both cardiac dysfunction and the altered β-adrenergic signaling system (catecholamine levels and signaling proteins) lead to a higher risk of lethal arrhythmia induced by β-adrenergic stimulation. This study contributes to the understanding of MetS’s repercussions on cardiac function and highlights the relevance of targeting MetS before its pathological progression towards cardiomyopathy.

## Figures and Tables

**Table 1:** Macroscopic parameters of hearts from control and MetS rats.

	Control	MetS	*p*
HW (g)	2.22 ± 0.07	2.48 ± 0.06	**0.0083**
RVW (g)	0.52 ± 0.03	0.56 ± 0.02	0.3165
LVW (g)	1.16 ± 0.06	1.27 ± 0.03	0.0813
HW/BW	0.48 ± 0.01	0.40 ± 0.01	**0.0009**
LVW/HW	0.52 ± 0.01	0.52 ± 0.01	0.8772
RVW/HW	0.24 ± 0.01	0.23 ± 0.01	0.5518
HW/TL (g/cm)	0.48 ± 0.02	0.54 ± 0.01	**0.0089**
LVW/TL (g/cm)	0.25 ± 0.01	0.28 ± 0.01	0.0870
RVW/TL (g/cm)	0.11 ± 0.01	0.12 ± 0.01	0.3237

Data are means ± SEMs from 13 (control) and 14 (MetS) animals. BW: body weight, HW: heart weight, RVW: right ventricle free wall weight, LVW: left ventricle, and TL: tibia length. The statistical analysis was performed with the Student’s unpaired *t*-test. Significant differences are highlighted in bold.

**Table 2:** Percentage change in cardiac hemodynamic parameters following β-adrenergic stimulation.

	Control	MetS	*p* Value
HR	31.00 ± 7.54%	38.96 ± 4.99%	0.3770
CO	92.90 ± 18.19%	92.65 ± 26.34%	0.9944
SV	46.09 ± 8.74%	39.60 ± 17.98%	0.7756
EF	50.64 ± 7.28%	65.79 ± 21.05%	0.5608
ESP	107.90 ± 34.35%	101.60 ± 22.80%	0.8752
EDP	−1.84 ± 11.75%	97.91 ± 88.68%	0.3571
LVDP	113.30 ± 32.77%	126.60 ± 19.37%	0.7182
+dP/dt	165.50 ± 25.51%	202.70 ± 39.50%	0.4885
−dP/dt	127.00 ± 31.78%	157.10 ± 35.55%	0.5545
τ	−34.54 ± 8.83%	−33.30 ± 4.61%	0.8955
Ea	46.60 ± 24.60%	61.36 ± 25.57%	0.6229

Data are expressed as the percentage of change over the basal condition in response to β-adrenergic stimulation. Values are means ± SEMs from 6 (control) and 8 (MetS) animals. The statistical analysis was performed with the Student’s unpaired *t*-test. HR: heart rate; CO: cardiac output; SV: stroke volume; EF: ejection fraction; ESP: end-systolic pressure; EDP: end-diastolic pressure; LVDP: left ventricular developed pressure, +dP/dt: maximum rate of pressure rise during systole; −dP/dt: maximum rate of pressure fall during diastole; τ: isometric relaxation constant; and Ea: arterial elastance.

## Data Availability

The datasets that support the findings of this study are available from the corresponding authors upon request.

## Supplementary Materials

The following supporting information can be downloaded at https://www.mdpi.com/article/10.3390/ijms26167989/s1.

## Abbreviations

The following abbreviations are used in this manuscript:

AC	Adenylyl cyclase
βAR	β-adrenergic receptor
BW	Body weight
CO	Cardiac output
CVD	Cardiovascular disease
Ea	Arterial elastance
EF	Ejection fraction
GPCR	G protein-coupled receptor
GRK	GPCR kinase
HDL-C	Cholesterol associated to high-density lipoproteins
HW	Heart weight
LV	Left ventricle
LVW	LV weight
LVFW	LV free wall
MetS	Metabolic syndrome
PKA	Protein kinase A
RV	Right ventricle
RVW	RV weight
RVFW	RV free wall
TG	Triglyceride
SV	Stroke volume
