# Supplementary material for: Altered β-Adrenergic System, Cardiac Dysfunction, and Lethal Arrhythmia in a Rat Model of Metabolic Syndrome
Source: Int J Mol Sci. 2025 Aug 19;26(16):7989. doi: 10.3390/ijms26167989 (PMC12386379; doi:10.3390/ijms26167989)
Supplement: Supplementary file 1 [file ijms-26-07989-s001.zip › 3. Figure S1_Evaluation of ultrastructural changes of the heart in MetS rats_18june2025.pdf]

Supplementary Figure 1

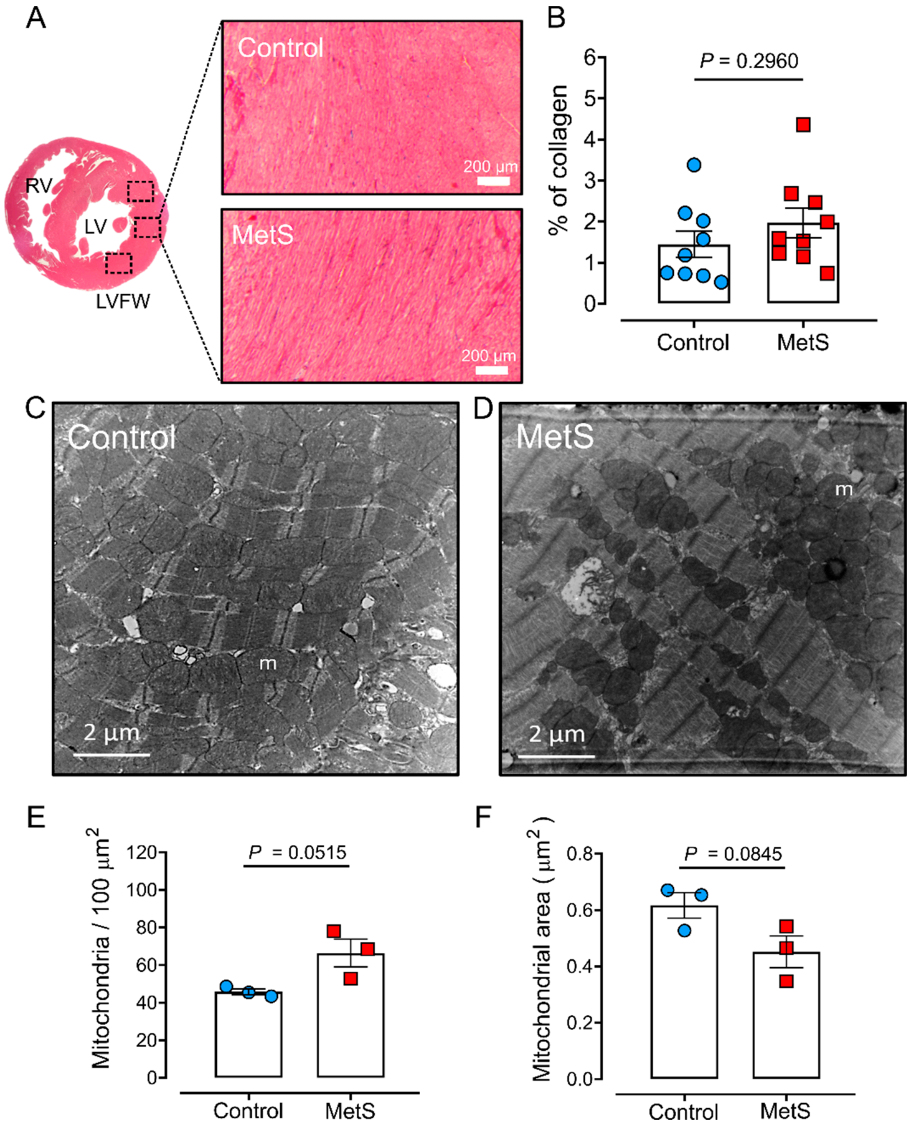

Figure S1. Evaluation of ultrastructural changes of the heart in MetS rats. A) Representative images of Masson's Trichrome staining of the left ventricle free wall (LVFW) of MetS and control animals, which data points represent induvial determinations. **B**) Quantification of the percentage of collagen deposition. Each dot represents the average of triplicate measurement from a single animal (N=9 animals per group). **C,D)** Representative microphotographs of control **(C)** and MetS **(D)** hearts (five microphotographs from a single animal, N=3 animals per group). **E,F**) Number (**E**) and area (**F**) of mitochondria (each dot represents the average the five determinations from a single animal, N = 3 rats per group). The statistical analyses were performed with Student's unpaired *t* test.
