# Supplementary material for: Altered β-Adrenergic System, Cardiac Dysfunction, and Lethal Arrhythmia in a Rat Model of Metabolic Syndrome
Source: Int J Mol Sci. 2025 Aug 19;26(16):7989. doi: 10.3390/ijms26167989 (PMC12386379; doi:10.3390/ijms26167989)
Supplement: Supplementary file 1 [file ijms-26-07989-s001.zip › 4. File S1_Uncropped blot_18june2025.pdf]

Supplementary File 1. Uncropped Western blots

Altered β-adrenergic system, cardiac dysfunction and lethal arrhythmia in a rat model of Metabolic Syndrome.

Original images for representative blots shown in Figure 7 and Figure 8 of manuscript.

1. Blots of the β_1AR and control load (GAPDH) proteins

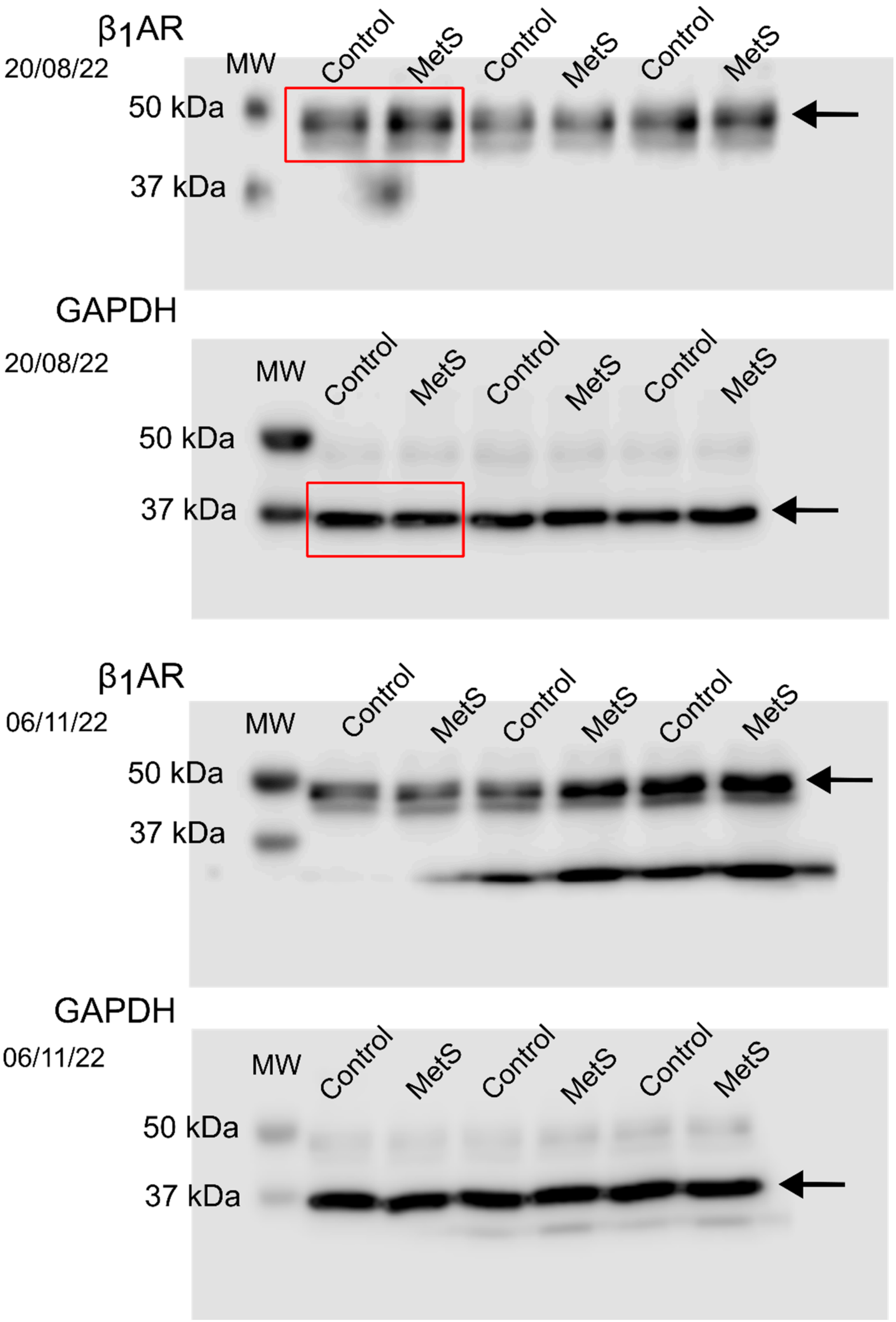

Blot 1 is shown in Figure 7C. The β_1AR was detected in LV tissue samples from control and MetS rats, loaded in alternating lanes. Representative blots are highlighted with a red rectangle. Molecular weight markers (MW) are displayed in the leftmost lane. The black arrow indicates the band corresponding to β_1AR. GAPDH was used as loading control. Equal amounts of protein (10 µg protein) were loaded.

2. Blots of the β2AR and control load (GAPDH) proteins

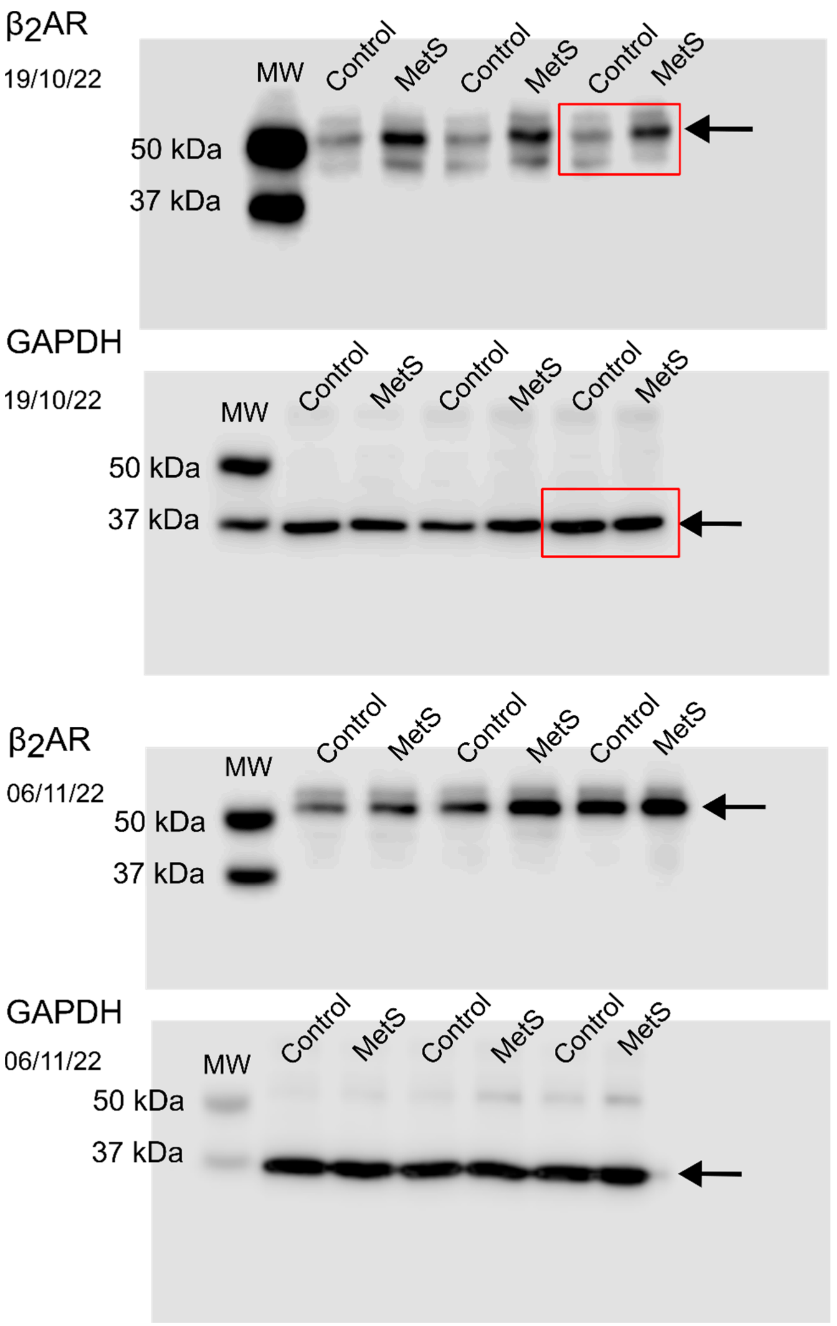

Blot 2 is shown in Figure 7D. The β2AR was detected in LV tissue samples from control and MetS rats, loaded in alternating lanes. Representative blots are highlighted with a red rectangle. Molecular weight markers (MW) are displayed in the leftmost lane. The black arrow indicates the band corresponding to β2AR. GAPDH was used as loading control. Equal amounts of protein (10 μg protein) were loaded.

3. Blots of the Gα_s and control load (GAPDH) proteins

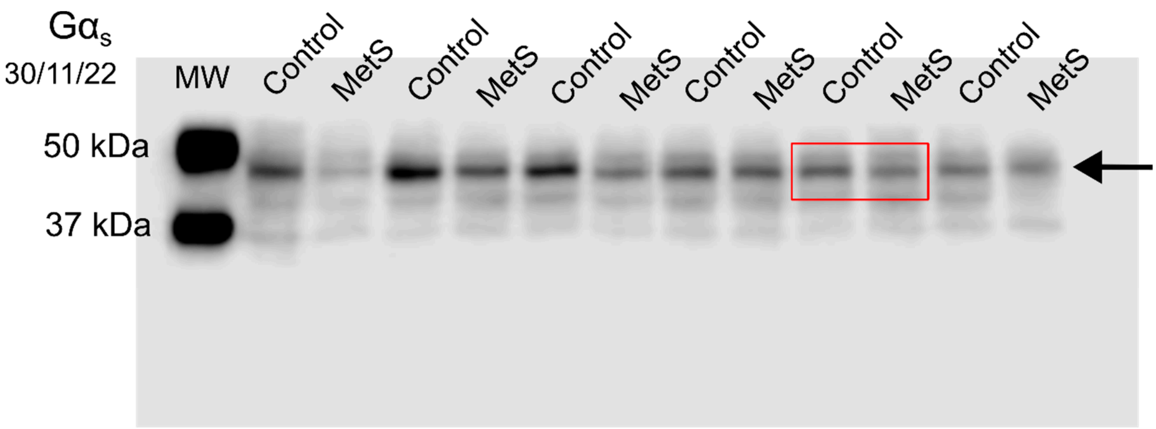

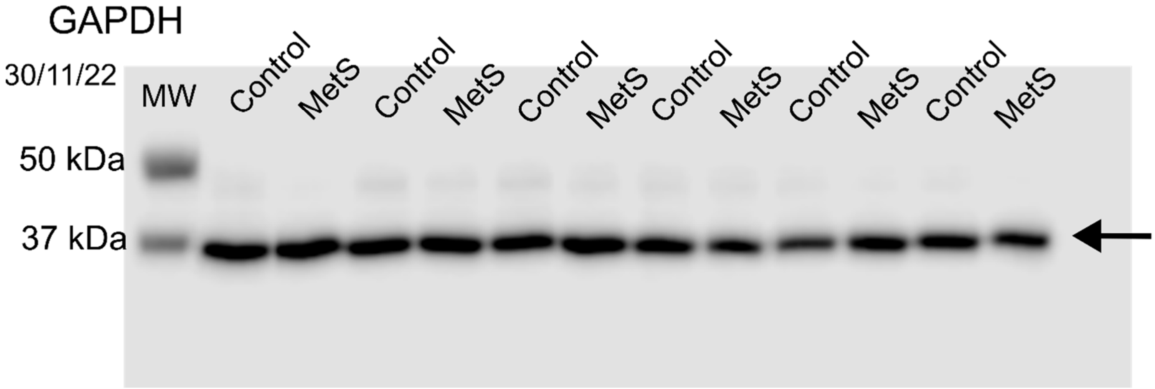

Blot 3 is shown in Figure 8A. The Gα_s was detected in LV tissue samples from control and MetS rats, loaded in alternating lanes. Representative blots are highlighted with a red rectangle. Molecular weight markers (MW) are displayed in the leftmost lane. The black arrow indicates the band corresponding to Gα_s. GAPDH was used as loading control. Equal amounts of protein (10 µg protein) were loaded.

4. Blots of the Gα$_i$ and control load (GAPDH) proteins

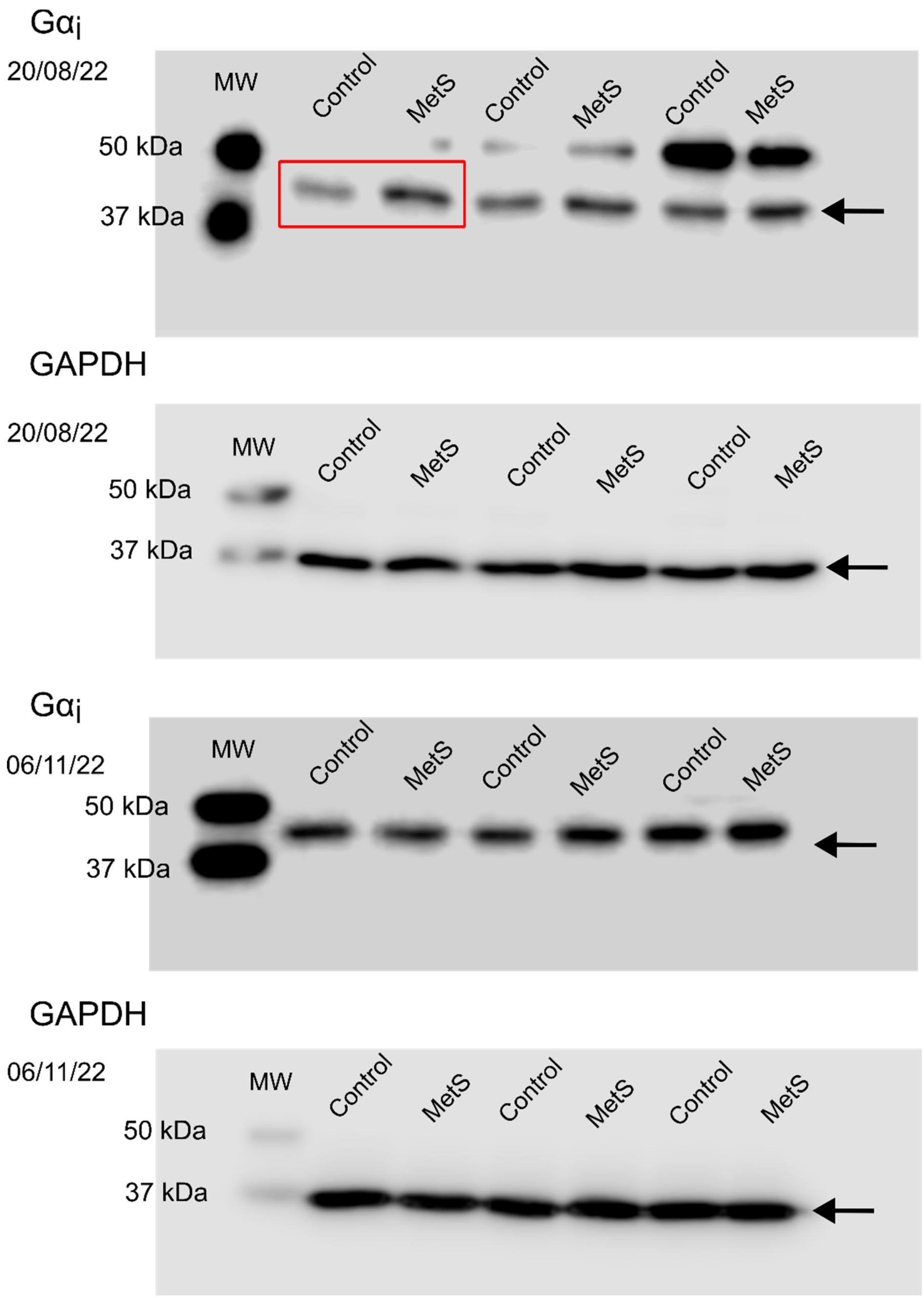

Blot 4 is shown in Figure 8A. The Gα$_i$ was detected in LV tissue samples from control and MetS rats, loaded in alternating lanes. Representative blots are highlighted with a red rectangle. Molecular weight markers (MW) are displayed in the leftmost lane. The black arrow indicates the band corresponding to Gα$_i$. GAPDH was used as loading control. Equal amounts of protein (10 μg protein) were loaded.

5. Blots of AC-V/VI and control load (GAPDH) proteins

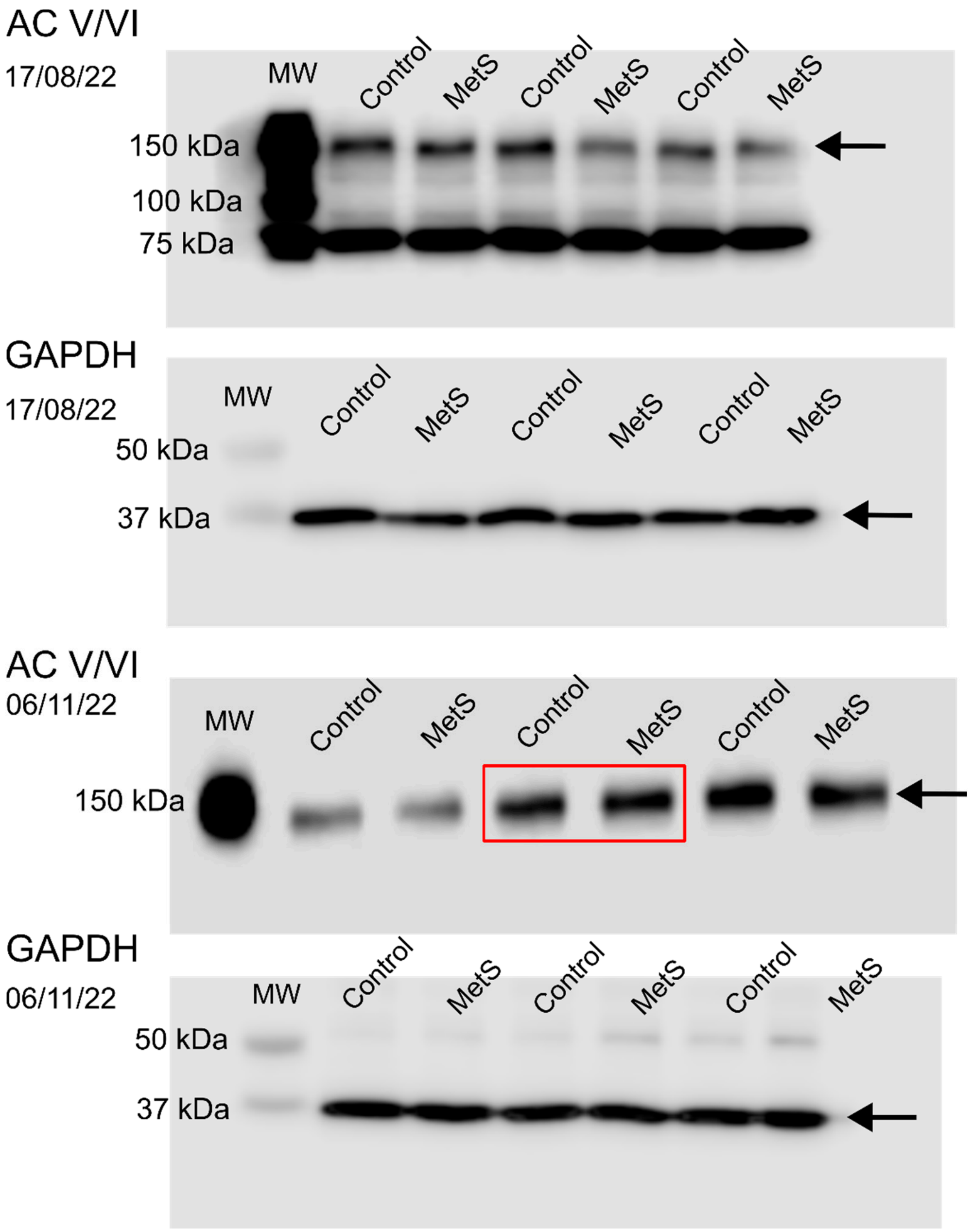

Blot 5 is shown in Figure 8A. The AC V/VI was detected in LV tissue samples from control and MetS rats, loaded in alternating lanes. Representative blots are highlighted with a red rectangle. Molecular weight markers (MW) are displayed in the leftmost lane. The black arrow indicates the band corresponding to AC V/VI. GAPDH was used as loading control. Equal amounts of protein (10 µg protein) were loaded.

6. Blots of PKA RIIα and control load (GAPDH) proteins

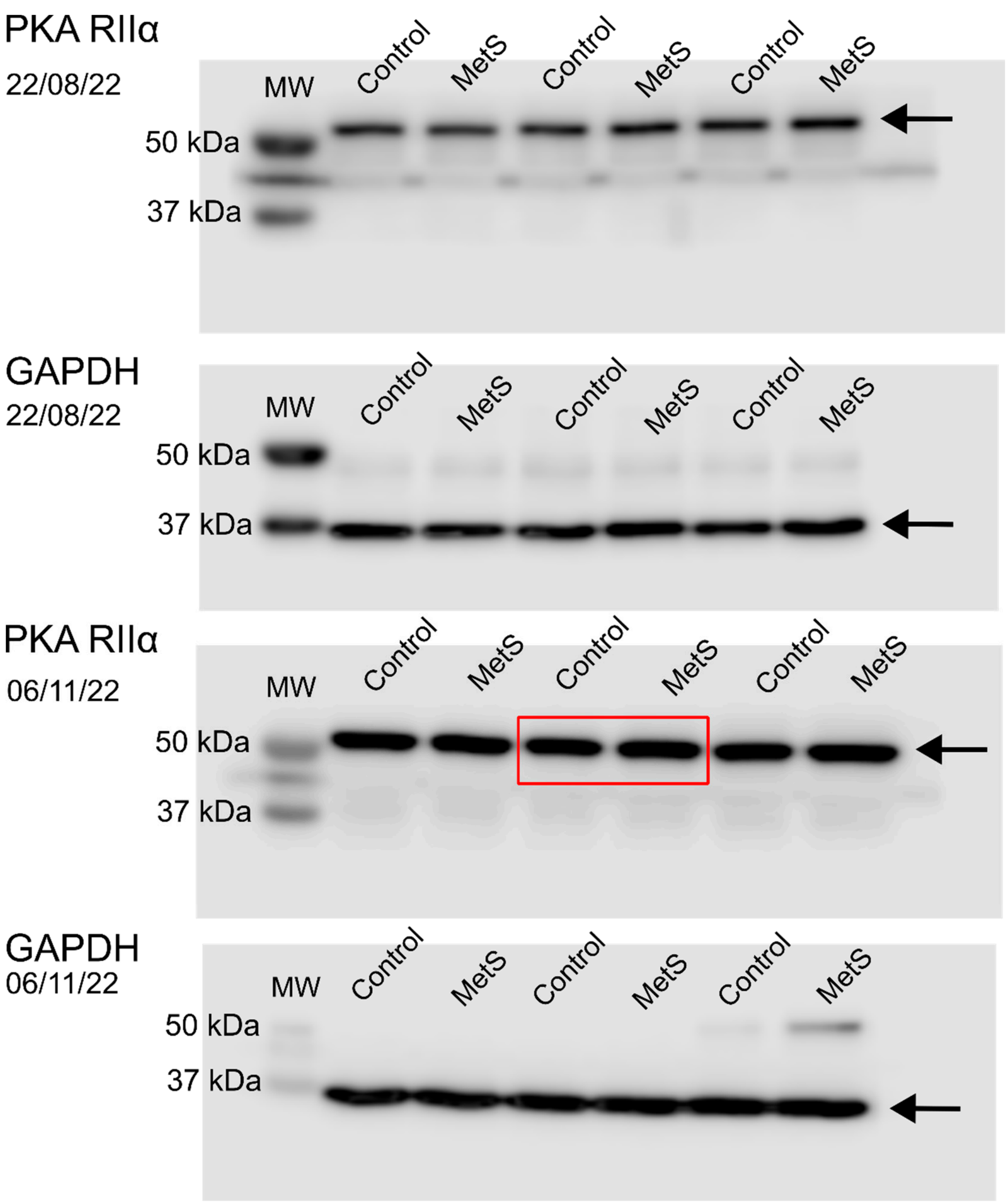

Blot 6 is shown in Figure 8A. The PKA RIIα was detected in LV tissue samples from control and MetS rats, loaded in alternating lanes. Representative blots are highlighted with a red rectangle. Molecular weight markers (MW) are displayed in the leftmost lane. The black arrow indicates the band corresponding to PKA RIIα. GAPDH was used as loading control. Equal amounts of protein (10 µg protein) were loaded.

7. Blots of β-arrestin 1 and control load (GAPDH) proteins

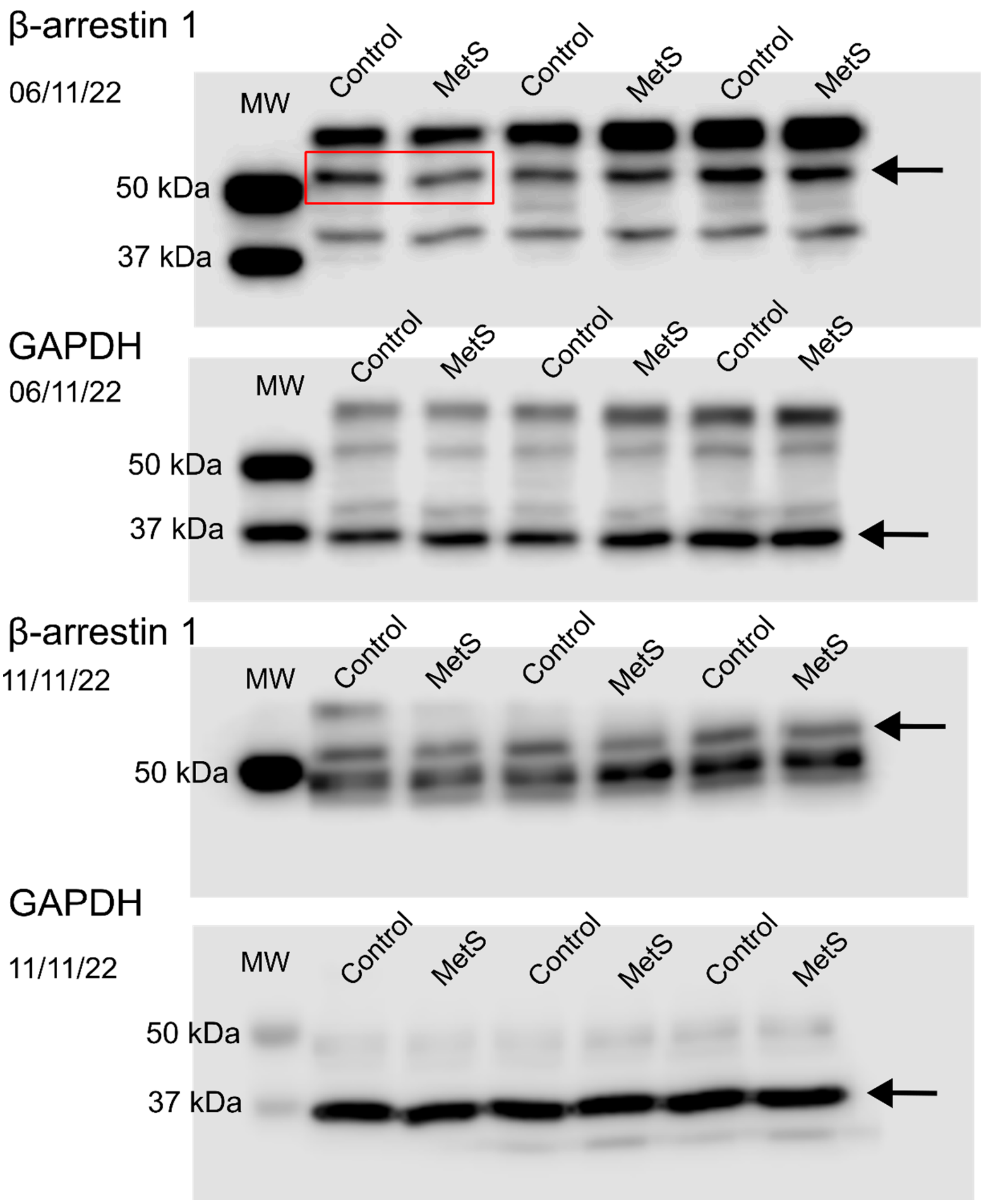

Blot 7 is shown in Figure 8A. The β-arrestin 1 was detected in LV tissue samples from control and MetS rats, loaded in alternating lanes. Representative blots are highlighted with a red rectangle. Molecular weight markers (MW) are displayed in the leftmost lane. The black arrow indicates the band corresponding to β-arrestin 1. GAPDH was used as loading control. Equal amounts of protein (10 µg protein) were loaded.

8. Blots of GRK2/3 and control load (GAPDH) proteins

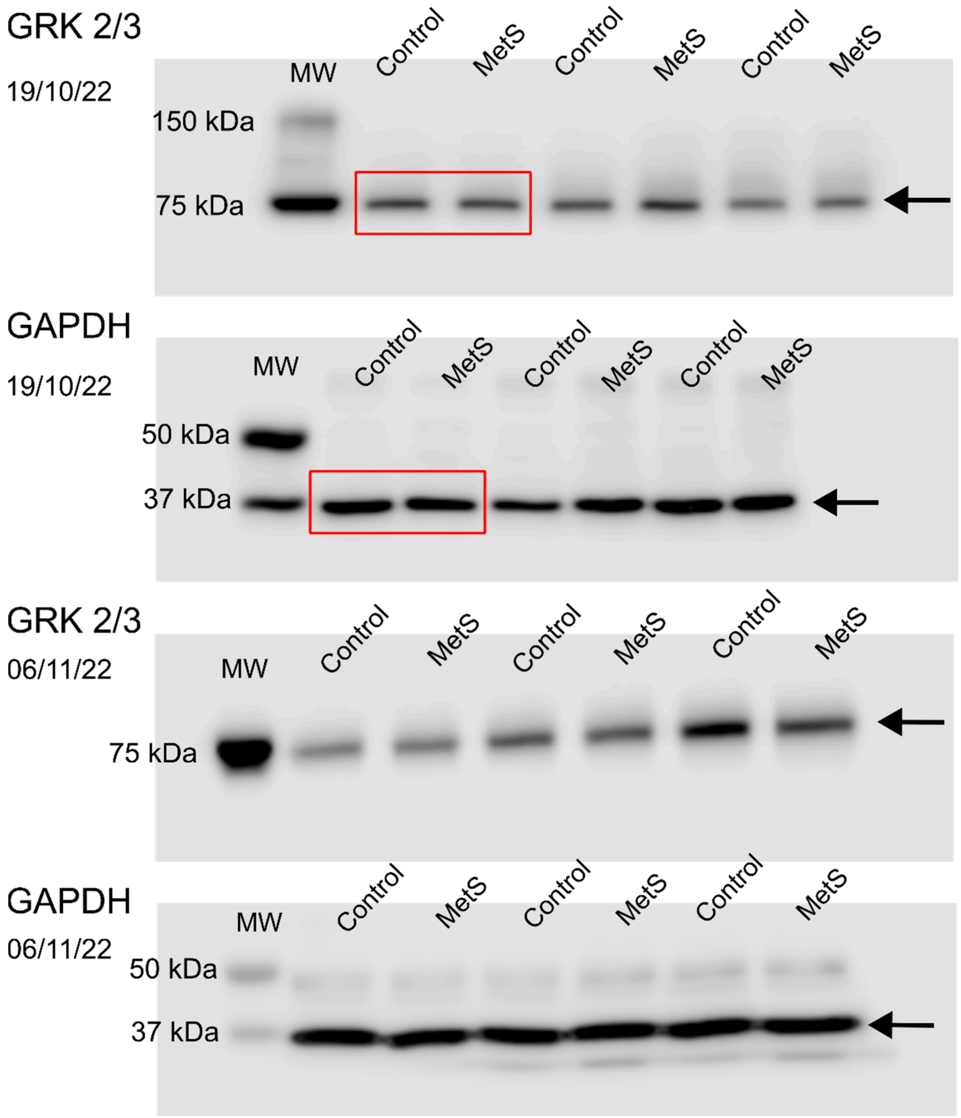

Blot 8 is shown in Figure 8A. The GRK 2/3 was detected in LV tissue samples from control and MetS rats, loaded in alternating lanes. Representative blots are highlighted with a red rectangle. Molecular weight markers (MW) are displayed in the leftmost lane. The black arrow indicates the band corresponding to GRK 2/3. GAPDH was used as loading control. Equal amounts of protein (10 µg protein) were loaded.

9. Blots of pCREB-Ser^{133} and CREB$_{Total}$ proteins

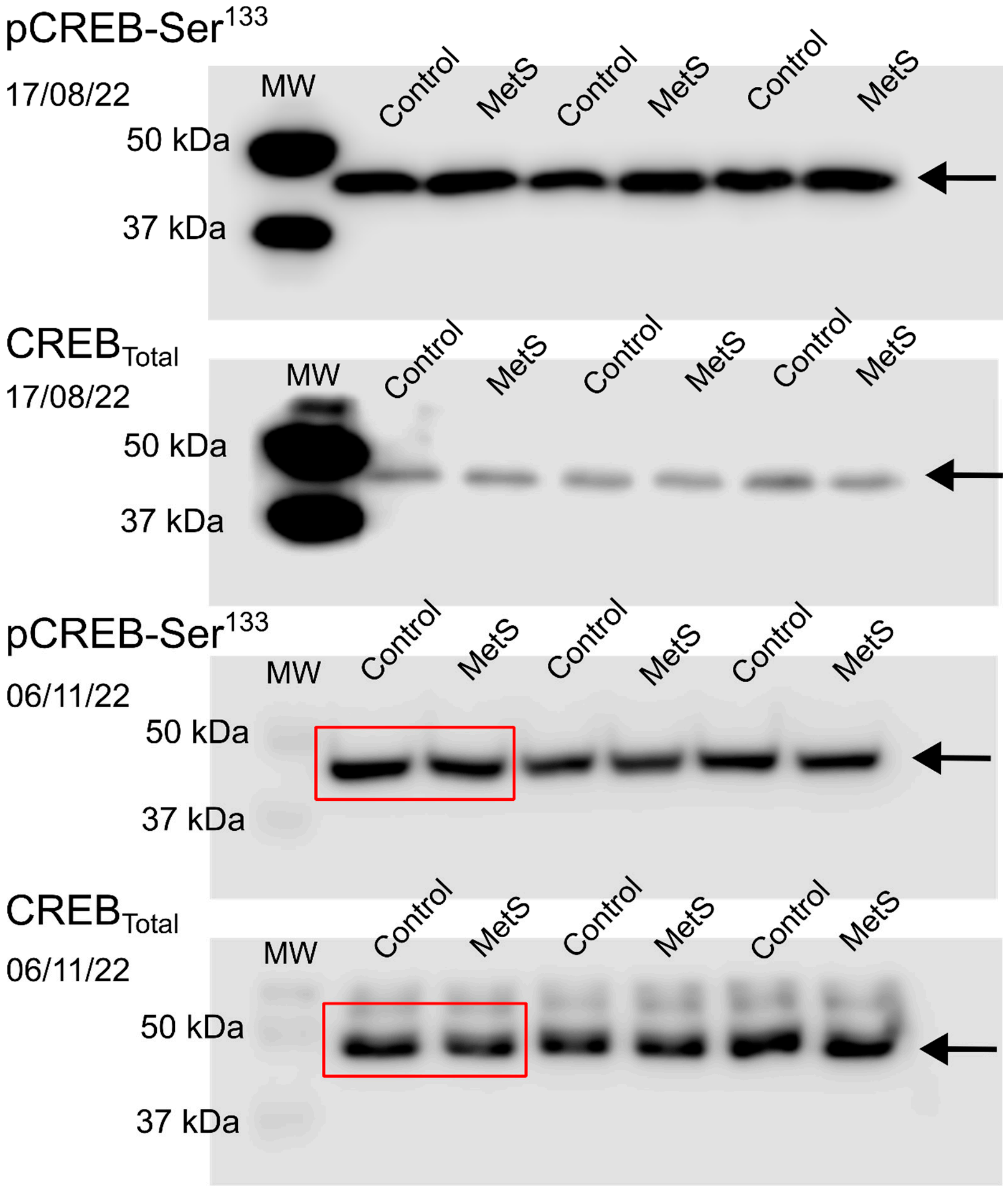

Blot 9 is shown in Figure 8H. pCREB-Ser^{133} and CREB$_{Total}$ were detected in LV tissue samples from control and MetS rats, loaded in alternating lanes. Representative blots are highlighted with a red rectangle. Molecular weight markers (MW) are displayed in the leftmost lane. The black arrow indicates the band corresponding to either pCREB-Ser^{133} and CREB$_{Total}$. Equal amounts of protein (10 μg protein) were loaded.
