# Supplementary material for: Altered β-Adrenergic System, Cardiac Dysfunction, and Lethal Arrhythmia in a Rat Model of Metabolic Syndrome
Source: Int J Mol Sci. 2025 Aug 19;26(16):7989. doi: 10.3390/ijms26167989 (PMC12386379; doi:10.3390/ijms26167989)
Supplement: Supplementary file 1 [file ijms-26-07989-s001.zip › 5. Table S1_Antibodies_18june2025.pdf]

Table S1. Antibodies employed in this study.

Protein	Dilution	Host	Company	Catalogue number
β_1AR	1:1000	Rabbit	ABClonal	A20818
β_2AR	1:1000	Rabbit	Badrilla	A-β2AR-25
AC V/VI	1:1000	Rabbit	Santa Cruz Biotechnology	sc-590
$G\alpha_s$	1:3000	Mouse	Santa Cruz Biotechnology	sc-135914
$G\alpha_i$ (α_{i1}, α_{i2}, α_{i3})	1:1000	Rabbit	Cell Signaling Technology	5290
PKA RIIα	1:1000	Rabbit	ABClonal	A1531
pCREB1-Ser^{133}	1:2000	Rabbit	ABClonal	AP0019
CREB1	1:1000	Rabbit	ABClonal	A10826
β-arrestin 1	1:1000	Mouse	BD Biosciences	610550
GRK 2/3	1:1000	Rabbit	Santa Cruz Biotechnology	sc-8329
GAPDH	1:50 000	Mouse	Ambion	AM4300
anti-rabbit IgG	1:10 000	Goat	MilliporeSigma Corp	401215
anti-mouse IgG	1:10 000	Goat	MilliporeSigma Corp	401315
